# IFNs Modify the Proteome of *Legionella*-Containing Vacuoles and Restrict Infection Via IRG1-Derived Itaconic Acid

**DOI:** 10.1371/journal.ppat.1005408

**Published:** 2016-02-01

**Authors:** Jan Naujoks, Christoph Tabeling, Brian D. Dill, Christine Hoffmann, Andrew S. Brown, Mareike Kunze, Stefan Kempa, Andrea Peter, Hans-Joachim Mollenkopf, Anca Dorhoi, Olivia Kershaw, Achim D. Gruber, Leif E. Sander, Martin Witzenrath, Susanne Herold, Andreas Nerlich, Andreas C. Hocke, Ian van Driel, Norbert Suttorp, Sammy Bedoui, Hubert Hilbi, Matthias Trost, Bastian Opitz

**Affiliations:** 1 Department of Internal Medicine/Infectious Diseases and Pulmonary Medicine, Charité University Medicine Berlin, Berlin, Germany; 2 MRC Protein Phosphorylation Unit, University of Dundee, Dundee, United Kingdom; 3 Max-von-Pettenkofer Institute, Ludwig Maximilian University, Munich, Germany; 4 The Department of Biochemistry and Molecular Biology, The University of Melbourne, Melbourne, Australia; 5 Integrative Metabolomics and Proteomics, Institute of Medical Systems Biology/Max-Delbrueck Center for Molecular Medicine, Berlin, Germany; 6 Max Planck Institute for Infection Biology, Berlin, Germany; 7 Department of Veterinary Pathology, Free University Berlin, Berlin, Germany; 8 Medizinische Klinik II, University Giessen and Marburg Lung Center, Justus-Liebig-University Giessen, Giessen, Germany; 9 The Department of Microbiology and Immunology, The University of Melbourne, Melbourne, Australia; 10 Institute of Medical Microbiology, University of Zurich, Zurich, Switzerland; University of São Paulo FMRP/USP, BRAZIL

## Abstract

Macrophages can be niches for bacterial pathogens or antibacterial effector cells depending on the pathogen and signals from the immune system. Here we show that type I and II IFNs are master regulators of gene expression during *Legionella pneumophila* infection, and activators of an alveolar macrophage-intrinsic immune response that restricts bacterial growth during pneumonia. Quantitative mass spectrometry revealed that both IFNs substantially modify *Legionella*-containing vacuoles, and comparative analyses reveal distinct subsets of transcriptionally and spatially IFN-regulated proteins. Immune-responsive gene (IRG)1 is induced by IFNs in mitochondria that closely associate with *Legionella*-containing vacuoles, and mediates production of itaconic acid. This metabolite is bactericidal against intravacuolar *L*. *pneumophila* as well as extracellular multidrug-resistant Gram-positive and -negative bacteria. Our study explores the overall role IFNs play in inducing substantial remodeling of bacterial vacuoles and in stimulating production of IRG1-derived itaconic acid which targets intravacuolar pathogens. IRG1 or its product itaconic acid might be therapeutically targetable to fight intracellular and drug-resistant bacteria.

## Author Summary

Numerous intracellular bacterial pathogens replicate in specialized vacuoles within macrophages. We systematically study the molecular mechanism and the impact of macrophage-intrinsic antibacterial defense. Using *L*. *pneumophila*, an important cause of pneumonia and model organism for intracellular bacteria, we found that type I and II interferons critically modify the proteome of bacterial vacuoles to restrict infection. We identify IRG1 and demonstrate a bactericidal activity of its metabolite itaconic acid on bacteria in their vacuole. Moreover, our study provides evidence for the impact of this cell-autonomous defense pathway in alveolar macrophages to restrict lung infection. We speculate that vacuolar IRG1 or its product itaconic acid could serve as future therapeutic targets to fight intracellular and drug-resistant bacteria.

## Introduction

Intracellular bacteria are major causes of morbidity and mortality. Upon infection, many intracellular pathogens establish intracellular membrane-bound compartments, where they resist lysosomal degradation and humoral immune responses [1]. As a result of co-evolution, host cells have in turn developed strategies to target the vacuoles or the bacteria inside in order to control infections [2,3]. Interferons (IFNs), which are classified into type I, II and III IFNs [4], are potent inducers of intracellular immunity in vertebrates [2]. They fulfill this function by activating transcription of partly overlapping sets of so-called IFN-stimulated genes (ISGs), several of which with antiviral or antibacterial activities. However, the exact functions of many ISGs remain unknown [2].

*Legionella pneumophila* is a frequent cause of severe pneumonia in humans and a model for investigating immune responses to intravacuolar bacteria. Upon infection, *L*. *pneumophila* is phagocytosed by alveolar macrophages, where *L*. *pneumophila* establishes a specialized replication vacuole, named the *Legionella*-containing vacuole (LCV). This process requires the Dot/Icm type IV secretion system (T4SS) which injects around 300 bacterial effector molecules into the host cytosol [5]. In replication-permissive cells, the LCV escapes fusion with lysosomes and instead recruits secretory vesicles from the endoplasmic reticulum (ER) as well as mitochondria [5–8].

In macrophages of C57BL/6 mice (and most other inbred strains), however, wild-type (wt) *L*. *pneumophila* is restricted by the NAIP5 inflammasome which detects bacterial flagellin and stimulates cell death as well as phagolysosomal maturation [9–13]. In contrast to wt bacteria, *L*. *pneumophila* lacking flagellin are not recognized by NAIP5 and are thus able to replicate in mouse macrophages. We and others recently demonstrated that *L*. *pneumophila* is additionally controlled by a cell-autonomous defense pathway that is activated by auto-/paracrine type I IFN signaling [14–19]. This defense pathway restricts the bacteria in their vacuole without preventing LCV formation or triggering lysosomal fusion [15].

In the present study, we systematically examined the antibacterial innate immune response to *L*. *pneumophila* infection and demonstrate that type I and II IFNs substantially alter the composition of bacterial vacuoles, induce production of bactericidal itaconic acid via IRG1, and restrict *L*. *pneumophila* replication in alveolar macrophages and lungs.

## Results

### IFNs are master regulators of gene expression upon *L*. *pneumophila* infection

**Fig 1:**
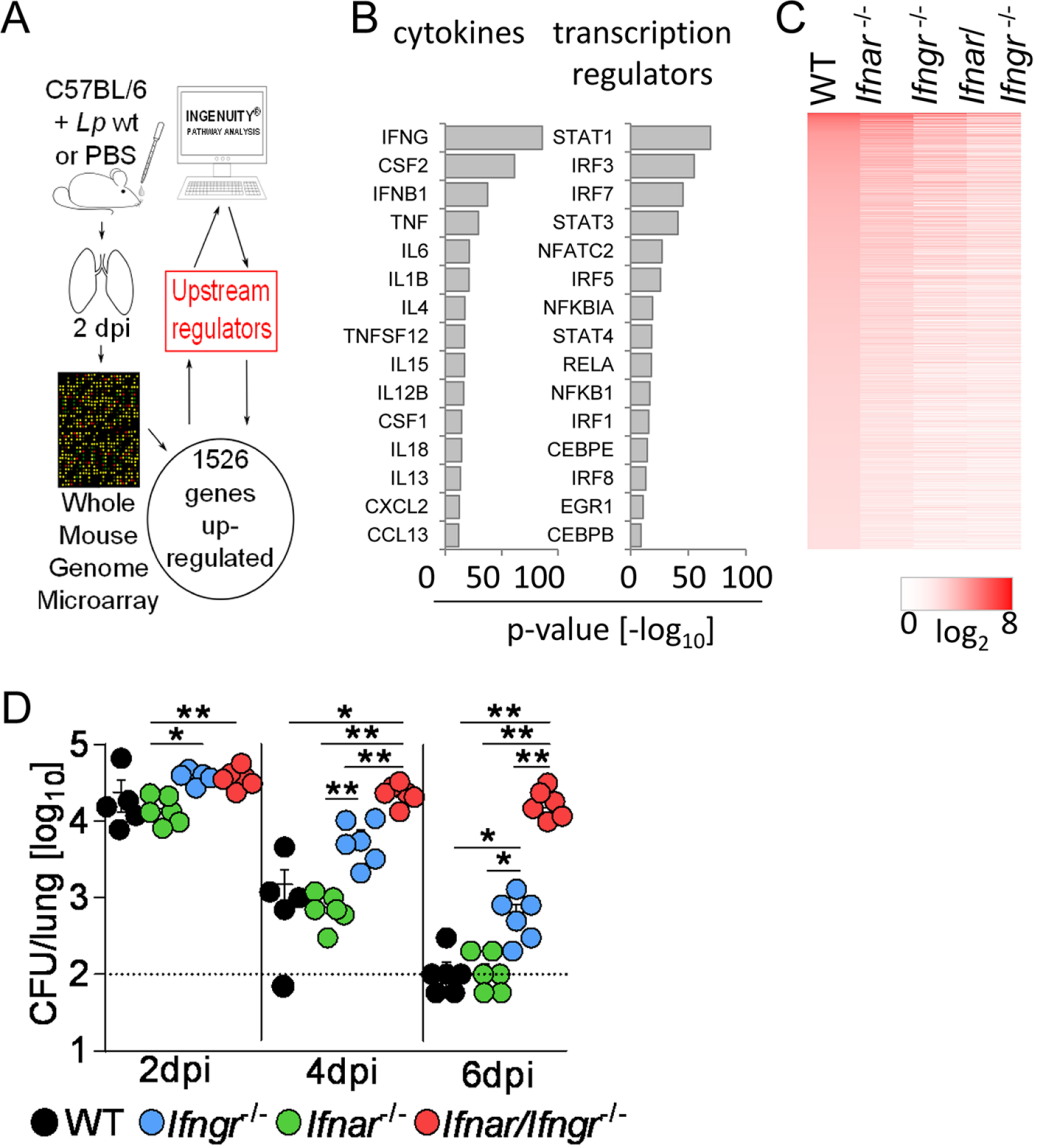
Type I and II IFNs are main regulators of gene expression during *L*. *pneumophila* infection. (A) Workflow for upstream regulator analysis. (B, C) WT (B) or WT, *Ifnar* ^-/-^, *Ifngr* ^-/-^ and *Ifnar*/*Ifngr* ^-/-^ mice (C) were infected with *L*. *pneumophila* or treated with PBS. Mice were sacrificed 2 d p.i., and microarray analysis performed from lung RNA. Up-regulated genes in *L*. *pneumophila*-infected WT mice compared to controls were analyzed for their predicted upstream regulators (B). Heat map of microarray analysis of infected versus PBS-treated mice for wild-type and respective knock-out strains (C). 5 mice per group, pooled for RNA extraction and subsequent analysis. (D) Bacterial loads in the lungs of *L*. *pneumophila* infected WT, *Ifnar* ^-/-^, *Ifngr* ^-/-^ and *Ifnar*/*Ifngr* ^-/-^ mice are depicted. Data represent mean + s.e.m. of 5–6 mice per group. Dotted line indicates lower detection limit, * p<0.05, ** p<0.01 (Kruskal-Wallis analysis of variance followed by Mann-Whitney U test with Bonferroni correction for multiple comparisons).

In order to identify master regulators of the innate immune response to intracellular bacteria, we compared gene expression in the lungs of *L*. *pneumophila*-infected and sham-treated C57BL/6 WT mice. We identified 1526 genes (S1 Dataset) that were induced upon infection. Upstream regulator analysis was performed with Ingenuity Pathway Analysis (IPA) (Fig 1A) and revealed that type I and II IFNs and their related transcription factors (e.g. STAT1, IRF3, IRF7) play a predominant role in controlling gene transcription in response to *L*. *pneumophila* infection (Fig 1B). This *in silico* prediction was confirmed by transcriptome analysis of *L*. *pneumophila*-infected *Ifnar* ^-/-^, *Ifngr* ^-/-^, *Ifnar*/*Ifngr* ^-/-^ mice, all of which showed a severely impaired transcriptional response compared to WT animals (Fig 1C, S1 Dataset).

To investigate the functional relevance of the type I and II IFNs for the antibacterial defense against *L*. *pneumophila*, we analyzed bacterial clearance following infection of WT, *Ifnar* ^-/-^, *Ifngr* ^-/-^ and *Ifnar*/*Ifngr* ^-/-^ mice. Whereas WT, *Ifnar* ^-/-^ and *Ifngr* ^-/-^ mice were able to clear or strongly reduce bacterial burdens by day 6 post infection (p.i.), bacterial loads remained high in *Ifnar*/*Ifngr* ^-/-^ mice (Fig 1D). This is in line with our previously published results from infections with *L*. *pneumophila* Δ*flaA* [15]. Together, these data indicate that type I and type II IFNs are critical regulators of early gene expression and the antibacterial innate immune response during *L*. *pneumophila* infection.

### IFNs restrict *L*. *pneumophila in vivo* via a CD11c^+^ cell-intrinsic mechanism

**Fig 2:**
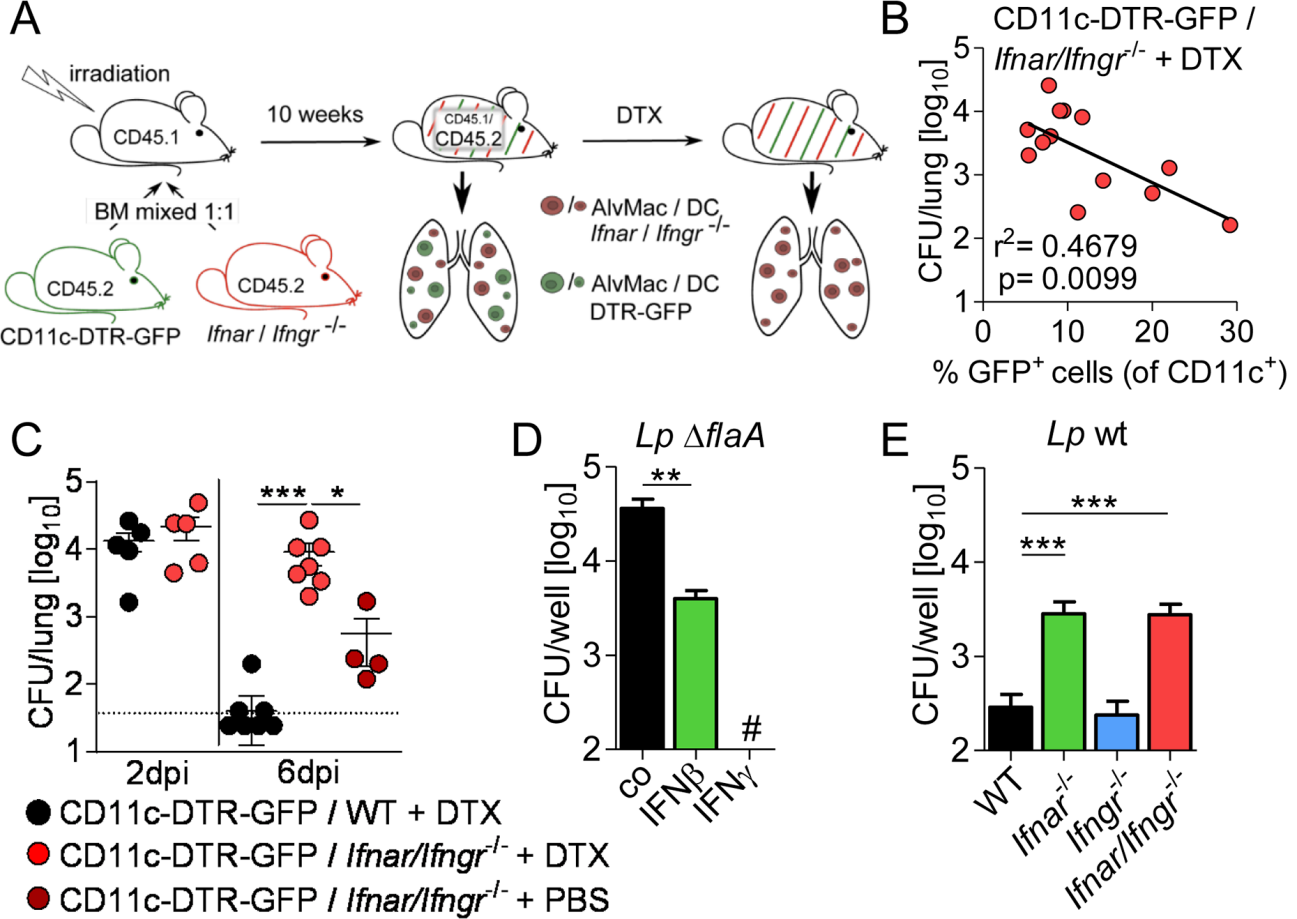
Type I and II IFNs restrict *L*. *pneumophila* infection through an alveolar macrophage-intrinsic mechanism. (A) Overview of generation of CD11c-DTR-GFP / *Ifnar*/*Ifngr* ^-/-^ mixed bone marrow chimeric mice followed by DTX mediated depletion of CD11c-DTR-GFP^+^ cells. (B) Frequency of remaining CD11c^+^ GFP^+^ wild-type cells was correlated to bacterial load in the lungs of CD11c-DTR-GFP / *Ifnar*/*Ifngr* ^-/-^ + DTX chimeras including all DTX-treated mice (13 mice) at 6 d p.i.. (C) Only mice with <10% GFP^+^ (of all CD11c^+^ cells) were considered for analysis and bacterial loads in the lungs of *L*. *pneumophila* infected mixed bone marrow-chimeric mice at indicated time points are shown. (D, E) Alveolar macrophages of WT, *Ifnar* ^-/-^, *Ifngr* ^-/-^ and *Ifnar/Ifngr* ^-/-^ mice were left untreated (E) or were treated with 50 U/ml IFNβ or IFNγ (D) 16–18 h prior to and during infection with *L*. *pneumophila* Δ*flaA* (D) or wt (E). Bacterial growth was determined by CFU counting after 72 h. Data represent 3 independent experiments done in triplicates. * p<0.05, ** p<0.01, *** p<0.001 (Pearson correlation (B), Kruskal-Wallis analysis of variance, Dunn’s multiple comparison (C) or Mann-Whitney U test (D, E)). # No bacteria were detected.

Alveolar macrophages, but not dendritic cells (DCs), are the primary cell type supporting *L*. *pneumophila* infection *in vivo* [20–22]. Therefore, we questioned whether an IFN-mediated alveolar macrophage-intrinsic defense pathway is relevant during *L*. *pneumophila* infection *in vivo*. To this end, we constructed a chimeric mouse model in which IFN signaling was selectively abrogated in CD11c^+^ cells, whereas at least 50% of all other hematopoietic cell types express the IFN receptors (Fig 2A). In the lung >90% of CD11c^+^ cells are alveolar macrophages and only a minority of pulmonary CD11c^+^ cells in steady state are DCs. CD45.1^+^ mice were irradiated and reconstituted with a 1:1 mixture of CD45.2^+^ bone-marrow cells from *Ifnar*/*Ifngr* ^-/-^ or WT and CD11c-DTR-GFP mice (expressing the diphtheria toxin receptor (DTR) under the control of the CD11c promoter). Repopulation was assessed to be >90% after 10 weeks (S1A Fig) and mice were subsequently infected with *L*. *pneumophila* wt. First, we analyzed all *L*. *pneumophila*-infected mice repopulated with *Ifnar/Ifngr* ^-/-^ and CD11c-DTR-GFP cells including those showing a weak depletion of CD11c^+^ GFP^+^ cells by diphtheria toxin (DTX) in the lung (S1B Fig). We observed a significant negative correlation between remaining CD11c^+^ GFP^+^ cells (expressing IFN receptors) and pulmonary bacterial load (Fig 2B). This correlation indicates that the number of IFN-responsive CD11c^+^ cells has a direct positive impact on bacterial clearance.

Second, we examined bacterial loads only in the bone-marrow-chimeric mice which showed a highly efficient DTX-mediated depletion of CD11c^+^ DTR-expressing GFP^+^ cells (with <10% remaining, S1 Fig). Strikingly, chimeric mice lacking the IFN receptors in CD11c^+^ cells (CD11c-DTR / *Ifnar*/*Ifngr* ^-/-^ + DTX) were unable to clear *L*. *pneumophila* wt infection (Fig 2C), and were thus comparable to *Ifnar*/*Ifngr* ^-/-^ mice (Fig 1D). In contrast, chimeric mice without depletion of IFNAR/IFNGR-expressing CD11c^+^ cells (CD11c-DTR / *Ifnar*/*Ifngr* ^-/-^ + PBS) showed a significant reduction of bacterial burdens (Fig 2C). Chimeric mice reconstituted with solely IFN-responsive cells (CD11c-DTR / WT + DTX) finally were able to clear the infection almost completely. Given that DCs do not support *L*. *pneumophila* growth [20,21], our data strongly suggest that IFNs induce alveolar macrophage-intrinsic effects to restrict intracellular infection.

In line with this conclusion, *L*. *pneumophila* Δ*flaA*, which is able to replicate in WT alveolar macrophages due to evasion of the NAIP5 inflammasome [9–12], is partially inhibited by IFNβ and completely blocked by IFNγ treatment (Fig 2D). Conversely, *Ifnar* ^-/-^ and *Ifnar*/*Ifngr* ^-/-^ alveolar macrophages supported replication of otherwise growth-restricted *L*. *pneumophila* wt (Fig 2E). These data indicate that endogenously produced type I IFNs control bacterial growth, whereas type II IFN is not relevant in this *ex vivo* model since alveolar macrophages produce no or only negligible levels of IFNγ [23]. Collectively, our data indicate that *L*. *pneumophila* lung infection is controlled by an IFN-dependent alveolar macrophage-intrinsic mechanism.

### IFNs restrict *L*. *pneumophila* in a largely iNOS- and cell death-independent fashion

To determine the molecular basis of how macrophages restrict *L*. *pneumophila* upon activation by IFNs, we made use of bone marrow-derived macrophages (BMMs), an easily available and frequently used cell model to study *L*. *pneumophila* infection [9–12,14,15]. As shown in alveolar macrophages (Fig 2D), treatment of BMMs with IFNβ or IFNγ restricted the growth of *L*. *pneumophila* Δ*flaA* (S2A and S2B Fig), which is in line with previous reports [14–16]. Importantly, treatment of BMMs with suboptimal doses of both cytokines alone or in combination resulted in comparable growth inhibition (S2C Fig) suggesting that type I and II IFNs might activate an identical intracellular restriction mechanism. Moreover, lack of responsiveness to endogenous IFNβ in *Ifnar* ^-/-^ BMMs resulted in replication of otherwise growth-restricted *L*. *pneumophila* wt, and further enhanced the growth of *L*. *pneumophila* Δ*flaA* (S2D Fig).

Type I IFNs have previously been reported to induce cell death via e.g. caspase-11-dependent pyroptosis or RIP3-dependent necroptosis [24,25]. In order to detect pyroptosis and necroptosis of infected BMMs, we measured cell viability by flow cytometry as a general readout for both types of cell death. The use of GFP-expessing *Legionella* allowed us to exclusively consider bacteria-harboring cells (S3A Fig). As expected, infection with *L*. *pneumophila* wt enhanced cell death compared to *L*. *pneumophila* Δ*flaA* as a consequence of NAIP5/NLRC4-dependent pyroptosis [10–12] (S3B, S3C and S3E Fig). However, cell death in *L*. *pneumophila* wt infected cells was not affected by the lack of IFNAR (S3B and S3C Fig), and was only marginally affected by IFNs upon *L*. *pneumophila* Δ*flaA* infection (S3B–S3D Fig). This indicates that IFNs can slightly enhance cell death in *L*. *pneumophila*-infected cells independently of the NAIP5 pathway. Moreover, cell death was completely independent of RIP3 (S3E Fig), and RIP3 as well as caspase-11 deficiency did not influence bacterial growth or its restriction by IFNs (S4A, S4B, S4D and S4E Fig). Another important restriction mechanism against intracellular bacteria is the production of nitric oxide (NO) via inducible NO synthase (iNOS) [26]. However, *L*. *pneumophila* wt and Δ*flaA* replication and IFN-mediated bacterial restriction were comparable in WT and iNOS-deficient macrophages (S4C and S4F Fig). Thus, neither cell death nor production of reactive nitrogen species by iNOS appear to be of critical importance for the IFN-mediated control of *L*. *pneumophila* infection.

### Subcellular quantitative proteomics reveal that type I and II IFNs substantially modify vacuolar protein composition

**Fig 3:**
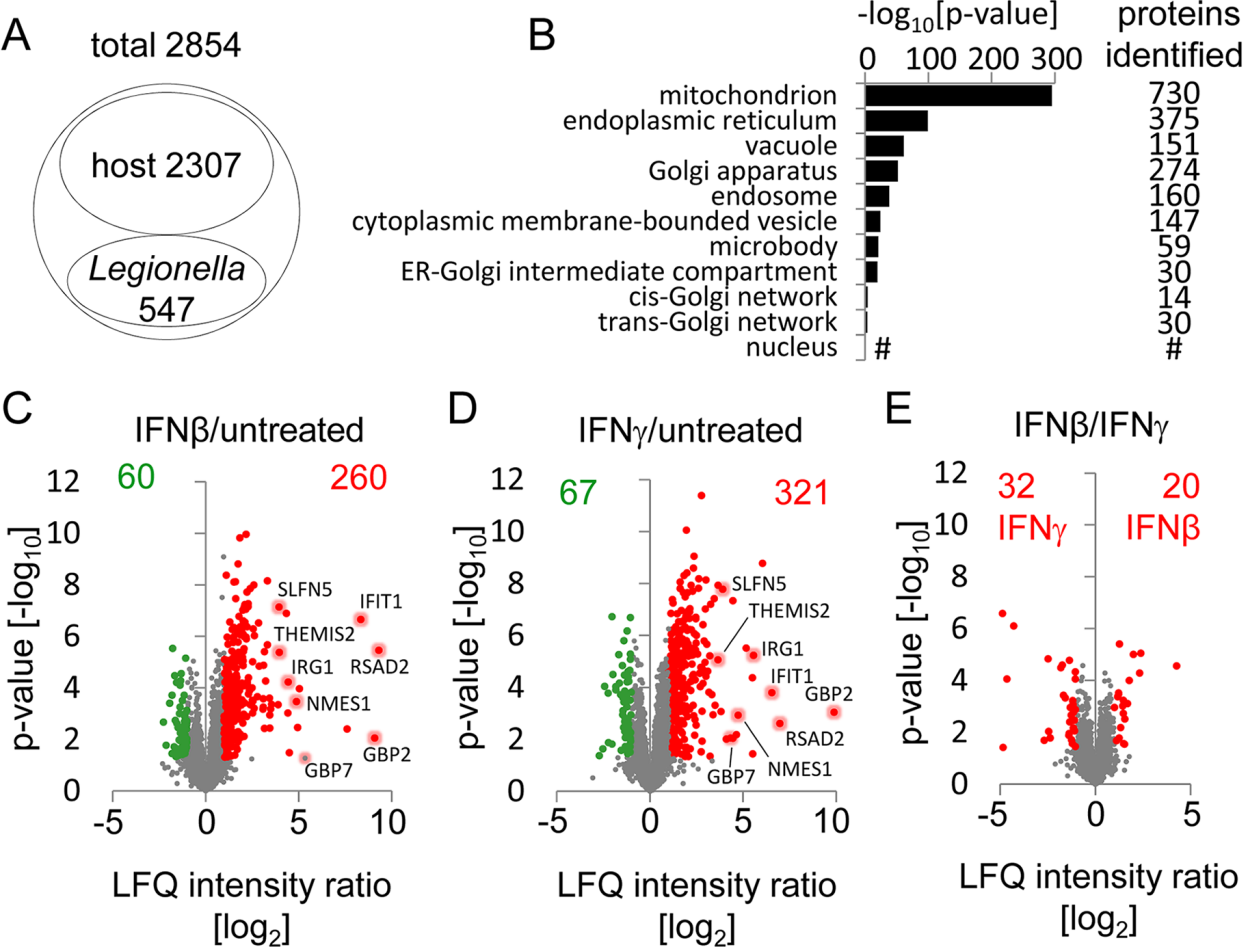
Type I and II IFNs alter the protein composition of LCVs. (A) Proteomic analysis of LCVs isolated 2 h p.i. from untreated BMMs infected with *L*. *pneumophila* Δ*flaA* detected 2854 proteins in all six replicates of which 2307 were identified as host- and 547 as *L*. *pneumophila*-derived. (B) GO enrichment analysis for overrepresented cellular components of the host proteins was done and overrepresented child terms of GO:0043231 *‘intracellular membrane-bounded organelle’* were extracted. Depicted are p-values for the indicated GO terms as well as the number of identified proteins annotated with each term; # no p-value for GO term *‘nucleus’* was computed. (C-E) Quantitative proteomic analysis of LCVs isolated 2 h p.i. with *L*. *pneumophila* Δ*flaA* from BMMs left untreated or treated with 50 U/ml IFNβ or IFNγ 16–18 h prior to and during infection. Volcano plots show unchanged proteins (grey) and proteins with a significant higher (red) or lower (green) abundance at LCVs from IFNβ- (C) or IFNγ- (D) treated BMMs compared to untreated cells, and direct comparison of IFNβ- versus IFNγ-treated samples (E). See also S6 Fig for detailed list of top 20 proteins for each condition. Proteomic analysis was done from 6 (untreated), 5 (IFNγ) and 4 (IFNβ) individual LCV isolations.

IFNs induce the expression of hundreds of ISGs, several of which possess antimicrobial activities. Since some antimicrobial ISGs have been associated with microbial vacuoles [2], we hypothesized that IFNs target antibacterial effector proteins to the LCV to restrict infection. In order to test this hypothesis in an unbiased and systematic fashion, we examined the proteome of *Legionella*-containing vacuoles (LCVs) in resting and IFN-activated macrophages 2 h post infection. First, resting macrophages were infected, LCVs were purified as previously described [27], and LCVs were analyzed by quantitative mass spectrometry. We identified 2307 proteins from the host and 547 from the bacterium in 6 of 6 samples of LCVs from untreated cells (Fig 3A, S2 Dataset). In order to determine the cellular origin of the identified proteins, we performed gene ontology (GO) enrichment analysis of the identified host proteins for cellular components (CC). This analysis revealed the highest significance values for the GO terms *‘membrane-bounded organelle’* and *‘intracellular membrane-bounded organelle’* as predicted cellular source of the identified proteins (S2 Dataset). Highest significance values were found for *‘mitochondrion’*, and *‘endoplasmic reticulum’* as predicted child terms of *‘intracellular membrane-bounded organelle’* (Fig 3B, S2 Dataset), reflecting both the ER-derived nature of the LCV as well as the previously reported close association of LCVs with mitochondria [5–8]. Additional GO enrichment analyses of biological processes (BP) indicated an enrichment of proteins involved in metabolic as well as transport and localization processes (S5 Fig, S2 Dataset).

**Fig 4:**
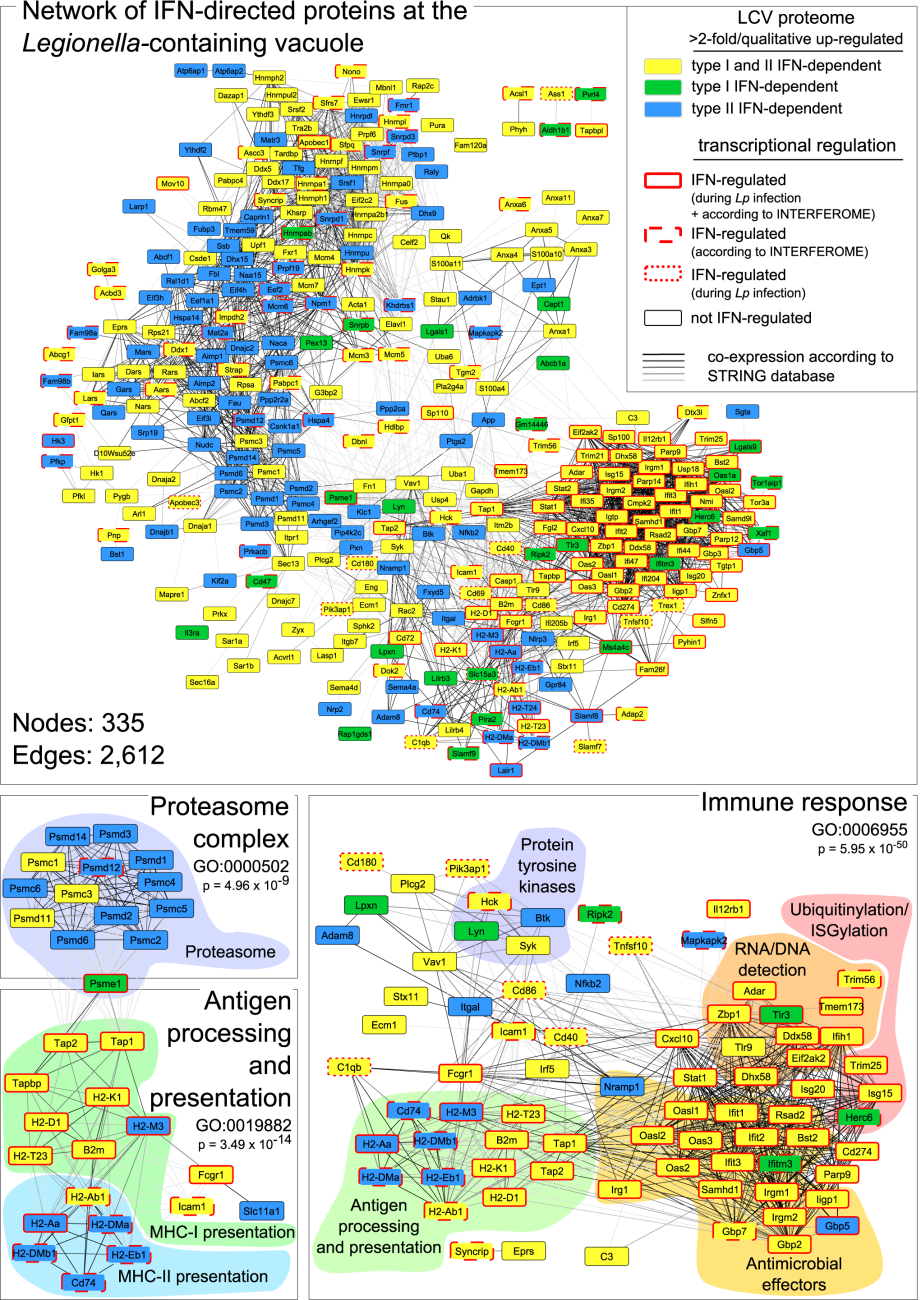
Integrated network analysis of IFN-regulated proteins of the LCVs. Proteins with higher abundance at LCVs from IFNβ- and/or IFNγ-treated compared to untreated cells were analyzed with the STRING database. The proteome data were further compared with the whole genome microarray data (S1 Dataset; genes > 2-fold higher expressed and p < 0.05 in infected WT vs. *Ifnar*/*Ifngr* ^-/-^ mice) and the INTERFEROME database to indicate molecules which are also transcriptionally regulated by IFNs. A GO enrichment analysis was performed for extracting significant subnetworks of a complex network composed of 335 nodes and 2,612 edges. Shown are subnetworks positively affected by IFNβ and/or IFNγ activation such as *‘immune response’*, *‘antigen processing and presentation’* and the *‘proteasome complex’*.

Macrophage activation by type I or II IFNs did not change the abundance of LCV marker proteins like ARF1 and SEC22b, or ER marker proteins, nor did it lead to an enrichment of endosomal or lysosomal proteins (Table 1), indicating that neither the LCV establishment is inhibited by IFNs nor do they trigger endo-lysosomal fusion. However, IFNβ or IFNγ treatment led to a significant (>2-fold) vacuolar enrichment of 260 or 321 proteins, respectively, and to a decreased vacuolar abundance of 60 or 67 proteins (Figs 3C, 3D, S6A–S6F, S6B, S6D, S6E and S3 Dataset). The direct comparison of LCV proteomes from IFNβ- or IFNγ-activated cells revealed rather minor differences with only a few proteins being differentially regulated (Figs 3E, S6C and S6F). Although we cannot exclude the possibility that some of the ISG product found on the vacuole could potentially only be a contaminant due to the massive up-regulation of ISGs in the cell, the data clearly show that IFNs substantially modify the LCV proteome. Computational analysis of all IFN-directed proteins at the LCV using the STRING database of known and predicted protein-protein interactions (http://string-db.org) generated a dense network of protein interactions, with many proteins being involved in immune response processes (Fig 4). These proteins included molecules contributing to microbial nucleic acid detection (e.g. TMEM173, also known as STING), ubiquitinylation/ISGylation (e.g. ISG15, TRIM25), antimicrobial defense (e.g. IRGM1, GBPs), and antigen processing/presentation. The comparison of the IFN-dependently LCV-enriched proteins with our transcriptome data (S1 Dataset) as well as the INTERFEROME database of ISGs [28] revealed distinct subsets of IFN-regulated proteins (Fig 4). Whereas several LCV-enriched proteins are also transcriptionally induced by IFNs and thus represent *bona fide* ISGs, others such as kinases Syk and Lyn or proteins of the proteasomal complex are not directly transcriptionally regulated but appear spatially affected by IFNs.

**Table 1:** Fold change in abundance of selected host proteins upon IFN-treatment identified by mass spectrometry on purified LCVs.

	IFNβ/untreated	IFNγ/untreated
**LCV marker**		
ARF1	1,7	1,7
RAB1a	1,3	1,1
RAB1a	1,0	1,1
SEC22b	1,1	1,0
**ER marker**		
Calreticulin	1,0	1,1
Calnexin	1,2	1,2
**endosome marker**		
RAB5a	1,8	1,8
RAB5b	1,5	1,7
RAB5c	1,6	1,7
RAB7a	1,2	1,2
RAB7b	1,2	1,1
**endosome/lysosome**		
vATPase subunit A	1,0	1,1
vATPase subunit C1	0,9	1,0
vATPase subunit G1	1,0	1,2
**lysosome marker**		
LAMP1	0,7	0,7
Cathepsin B	0,4	0,4
Myeloperoxidase	0,6	0,7

### IRG1 is a major *L*. *pneumophila* restricting factor

**Fig 5:**
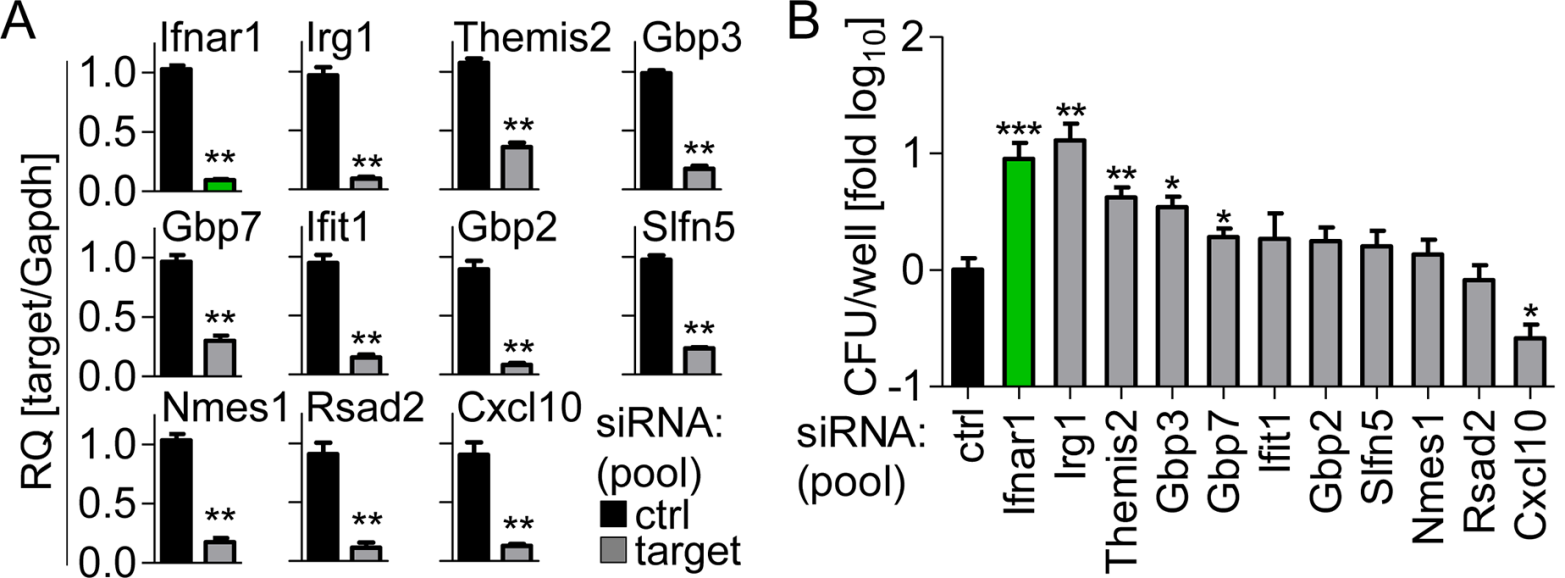
RNAi screen identifies IRG1 as crucial *L*. *pneumophila* restricting factor. (A, B) BMMs were transfected with control siRNA or a pool of two siRNAs per gene 24 h prior to infection and infected with *L*. *pneumophila*. Expression of targeted genes was assessed 24 h p.i. by qRT-PCR (A), and CFUs were counted 72 h p.i. (B). Data are mean + s.e.m. of 2 (A) or 4 (B) independent experiments done in triplicates. * p<0.05, ** p<0.01, *** p<0.001, no indication if not significant (Mann-Whitney U test).

In order to identify new IFN-regulated proteins possessing antibacterial activity against *L*. *pneumophila* we decided to examine proteins that were most strongly targeted to the LCV by both IFNs (Fig 3C and 3D) for their roles in restricting *L*. *pneumophila* growth. BMMs were first transfected with a pool of two siRNAs for each of our candidate molecules as well as IFNAR as a control, and efficient gene silencing was verified (Fig 5A). We found that silencing the expression of IRG1 enhanced replication of *L*. *pneumophila* to a similar extent as silencing of IFNAR, whereas knock-down of THEMIS2, GBP3, and GBP7 only slightly increased bacterial growth (Fig 5B). We thus decided to focus on IRG1.

**Fig 6:**
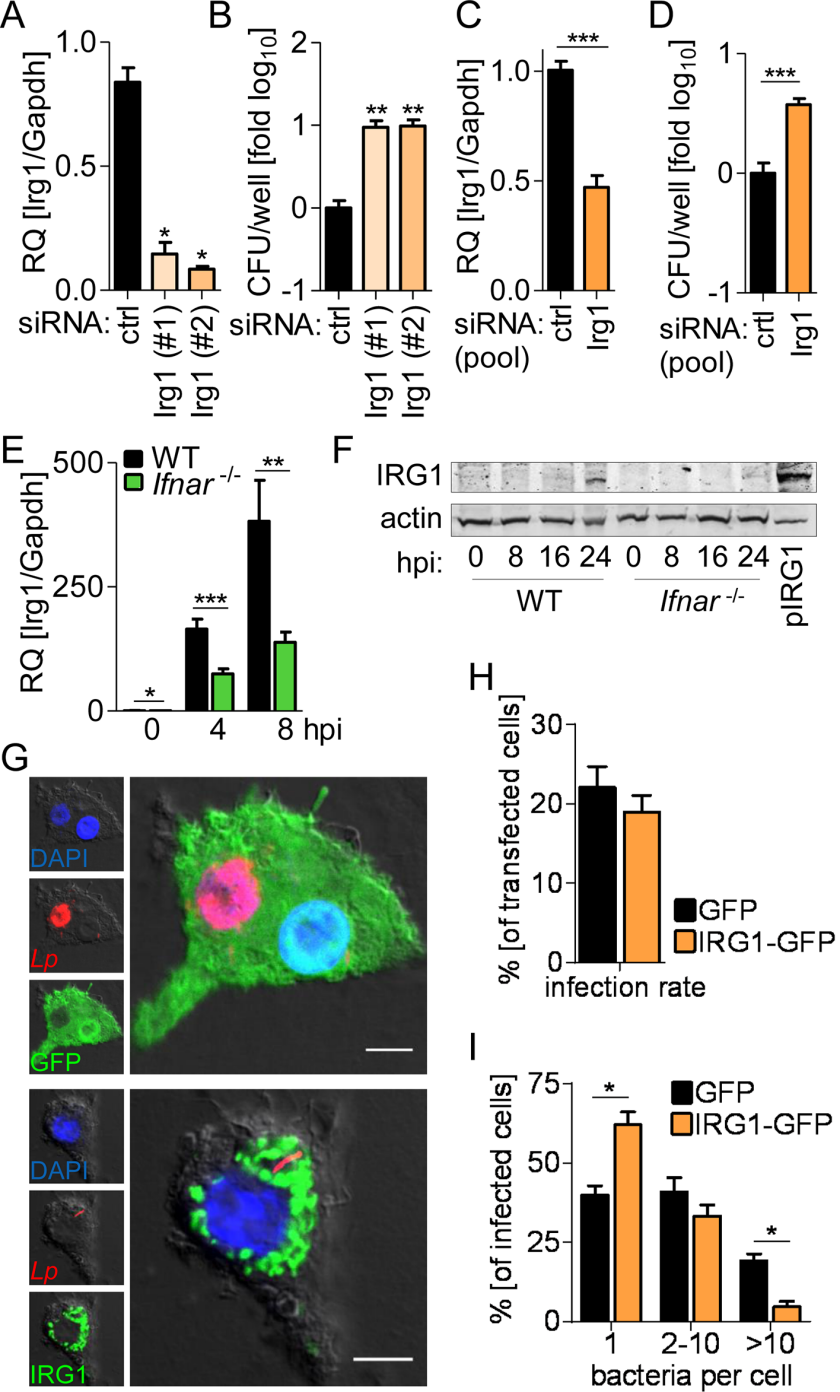
IRG1 is regulated by IFNs and restricts *L*. *pneumophila* within their vacuoles. (A-D) BMMs (A, B) or alveolar macrophages (C, D) were transfected with control or two IRG1 siRNAs separately (A, B) or pooled (C, D), infected with *L*. *pneumophila*, *Irg1* expression was assessed 24 h p.i. (A, C), and CFUs were counted 72 h p.i. (B, D). (E, F) *Irg1* gene and IRG1 protein expression in WT and *Ifnar* ^-/-^ BMMs upon infection with *L*. *pneumophila* was determined at indicated time points by qRT-PCR (E) and western blot (F). Cells overexpressing IRG1-Myc-DDK (pIRG1) were loaded as a positive control (F). (G-I) *Ifnar* ^-/-^ BMMs overexpressing IRG1-GFP or GFP only were infected with DsRed-expressing *L*. *pneumophila* (red). Cells were fixed 24 h p.i., representative images taken (G), and overall infection rate (H) as well as number of intracellular bacteria per cell determined by manual counting (I). (G) Scale bars indicate 5 μm. Data are mean + s.e.m. of 2 (A, B) or 4 (E) independent experiments done in triplicates or 3 independent experiments done in quadruplicates (C, D). * p<0.05, ** p<0.01, *** p<0.001, no indication if not significant (Mann-Whitney U test). (F) Representative blot of 3 independent experiments. (H, I) > 100 GFP- / IRG1-GFP-expressing cells were counted manually for intracellular bacterial numbers, data represent mean + s.e.m. of 2 independent experiments done in duplicates * p<0.05, no indication if not significant (Mann-Whitney U test).

Each IRG1-siRNA sequence was also effective in inhibiting their target gene expression (Fig 6A) and in increasing bacterial replication when used individually (Fig 6B). To demonstrate that the IRG1-mediated bacterial restriction is also relevant in primary alveolar macrophages we silenced IRG1 expression by siRNA (Fig 6C), which led to a significantly increased *L*. *pneumophila* growth in these cells compared to control cells (Fig 6D).

We found that IRG1 was strongly induced in BMMs by IFNβ and IFNγ treatment (S7 Fig), confirming its status as a *bona fide* ISG. Moreover, IRG1 expression was induced upon *L*. *pneumophila* infection at both transcriptional and protein levels, which strongly relied on the endogenous type I IFN signaling (Fig 6E and 6F). Strikingly, overexpression of IRG1 in *Ifnar* ^*-/-*^ cells restricted intravacuolar growth of *L*. *pneumophila*, while the percentage of infected cells was not influenced (Fig 6G–6I). Thus, IRG1 is regulated by IFNs, and restricts replication of *L*. *pneumophila* within the LCV.

### IRG1 localizes to mitochondria, which closely associate with the LCV

**Fig 7:**
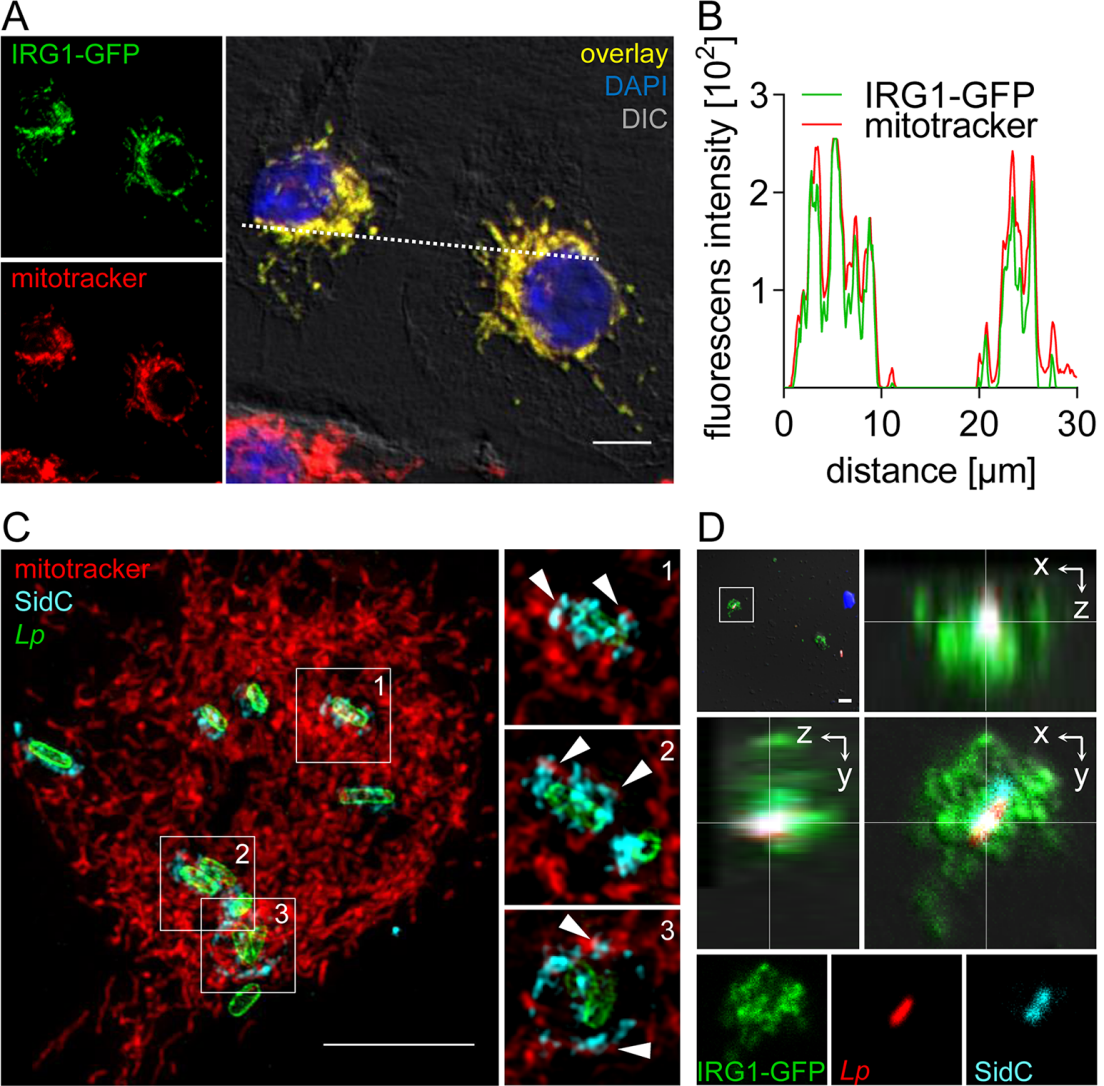
Mitochondrial IRG1 closely associates with LCVs. (A, B) *Ifnar* ^-/-^ BMMs overexpressing IRG1-GFP (green) were stained with mitotracker (red), fixed and nuclei were visualized by DAPI (blue) staining. Images represent maximum intensity projections of z-stacks of 10 individual 1.4 μm thick sections. (B) Fluorescence intensity profiles of IRG1-GFP and mitotracker along the dotted line in (A) are depicted. (C) Super-resolution fluorescent image generated by structured illumination microscopy of BMMs stained with mitotracker (red) and subsequently infected with *L*. *pneumophila* for 2 h. Bacteria and LCVs were visualized in fixed cells by *L*. *pneumophila* (green) and SidC (LCV-located *L*. *pneumophila*-protein; cyan) staining, respectively. Large overview image represents maximum intensity projection of a z-stack of 30 individual 0.126 μm thick sections. Enlarged sections in the left panel depict one single confocal plane for each selected LCV according to numbers in the overview image. White arrowheads point towards close associations of mitochondria with the LCV membrane. (D) *Ifnar* ^-/-^ BMMs overexpressing IRG1-GFP were infected with DsRed-expressing *L*. *pneumophila* wt (red). 2 h p.i. cells were homogenized and LCVs and nuclei were visualized by SidC (LCV-located *L*. *pneumophila*-protein; cyan) and DAPI (blue) staining. Framed area from the upper left overview image is shown as orthogonal view of a z-stack of 8 individual 0.68 μm thick sections and as single channels for depicted section. (A, C, D) Scale bars indicate 5 μm.

In agreement with previous studies in RAW264.7 macrophages [29], IRG1 showed a mitochondrial localization (Fig 7A and 7B). Moreover, super-resolution fluorescence microscopy demonstrated that mitochondria were distributed throughout the cell and closely associated with LCVs (Fig 7C). As viewed by time-lapse fluorescence microscopy, mitochondria moved very dynamically within living cells, although single mitochondria appeared to stay in close proximity to intracellular *L*. *pneumophila* for at least 1 h, most likely attached to the LCV membrane (S8 Fig and S1 Video). In order to directly evaluate whether the mitochondria-localized IRG1 associates with the LCV, we visualized homogenized *Legionella*-infected IRG1-GFP-overexpressing cells by fluorescence microscopy, and found that LCVs are surrounded by IRG1 (Fig 7D). In summary, these data confirm that overexpressed IRG1 localizes to mitochondria and that the latter are in close contact with LCVs. In absence of a specific antibody we could not obtain sufficient staining of endogenous IRG1. Nevertheless, our data strongly suggest that IFN signaling stimulates up-regulation of endogenous IRG1 within mitochondria, which results in its close association of IRG1 with LCV.

### IRG1 mediates production of the metabolite itaconic acid, which is broadly bactericidal

**Fig 8:**
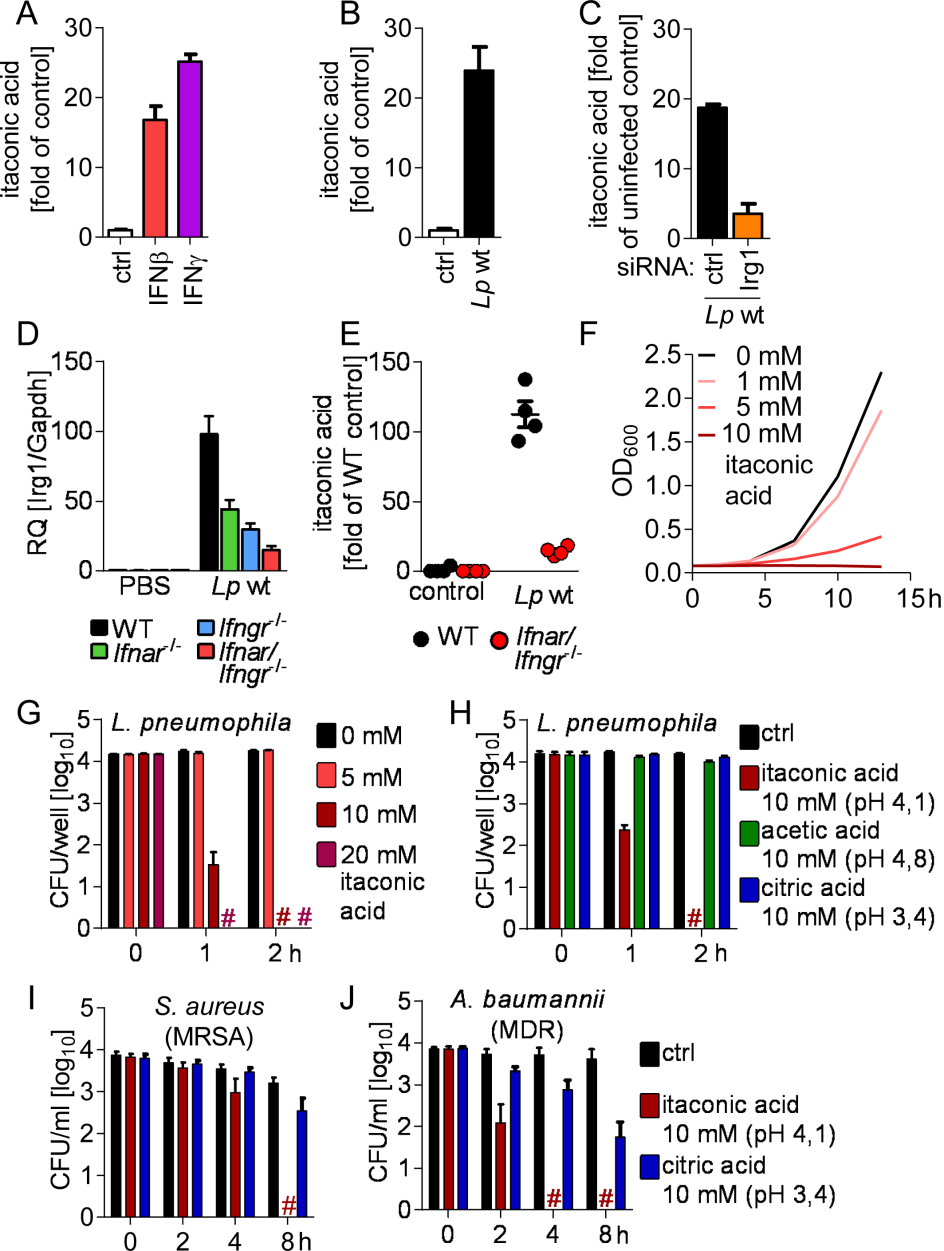
IRG1 mediates production of itaconic acid, which is bactericidal against *L*. *pneumophila* as well as multidrug-resistant Gram-positive and -negative bacteria. (A-C) Intracellular levels of itaconic acid were measured by GC-MS in BMMs after stimulation with 50 U/ml IFNβ or IFNγ for 16–18 h (A), infection with *L*. *pneumophila* for 24 h (B), and in BMMs transfected with siRNAs 24 h prior infection and infected with *L*. *pneumophila* for 24 h (C). (D, E) Irg1 expression (D) and itaconic acid levels (E) in lungs of *L*. *pneumophila*-infected or control mice measured by qRT-PCR or GC-MS, respectively, 2 d p.i.. (F) Itaconic acid was added to *L*. *pneumophila* in liquid culture and bacterial growth was assessed. (G-J) *L*. *pneumophila*, *S*. *aureus* (MRSA) and *A*. *baumannii* (MDR) were incubated in PBS containing itaconic acid or related acids, and numbers of viable bacteria were determined by CFU counting. (A-C) Data represent mean + s.e.m. of 3 (A) or 4 (B, C) experiments, done in sextuplicates pooled for GC-MS analysis. (D, E) Data represent mean + s.e.m. of 4 (E) or 5 (D) mice per group. (F) Representative graph of 3 independent experiments. (E, F) Data represent mean + s.e.m. of 2 (G) or 3 (H-J) experiments done in triplicates. # No viable bacteria were detected.

IRG1 has recently been described as an enzyme catalyzing the production of itaconic acid, which was found to exert bacteriostatic effects on *Mycobacteria* and *Salmonella* in liquid cultures [30], but the mechanisms that regulate this pathway and its relevance for infections remained incompletely understood. Subsequently, another study found that IRG1 mediates production of mitochondrial ROS (mROS) [31]. We found that mROS is produced in *Legionella*-infected macrophages by a largely IRG1- and IFNAR-independent mechanism (S9A and S9B Fig). In contrast, metabolic analyses of BMMs via gas chromatography-mass spectrometry (GC-MS) revealed that IFNs as well as *L*. *pneumophila* stimulate the production of itaconic acid (Fig 8A and 8B), whereas gene-silencing of IRG1 strongly reduced its production (Fig 8C). In line with the robust expression of IRG1 upon *L*. *pneumophila* infection *in vivo* (Fig 8D), GC-MS measurements revealed a strong production of itaconic acid also in *L*. *pneumophila*-infected mouse lungs (Fig 8E). Notably, IRG1 expression as well as itaconic acid production *in vivo* were largely dependent on functional IFN signaling (Fig 8D and 8E).

Next, we assessed the antibacterial potential of itaconic acid on *L*. *pneumophila*. In line with previous findings for *M*. *tuberculosis* and *S*. *enterica* [30], we found itaconic acid had an inhibitory effect on *L*. *pneumophila* growth in liquid culture (Fig 8F), however, at concentrations previously found insufficient to restrict bacterial growth. Importantly, itaconic acid, but not related organic acids, was capable of killing *L*. *pneumophila* as well as multidrug-resistant Gram-positive and -negative isolates (Fig 8G–8J) at concentrations that have recently been measured in activated RAW264.7 macrophages [30]. We thus conclude that IRG1 restricts *L*. *pneumophila* in their LCVs in macrophages through catalyzing the production of the broadly bactericidal metabolite itaconic acid.

## Discussion

IFNs execute antimicrobial functions by stimulating the expression of hundreds of ISGs. Recent large-scale examinations of ISGs have shed light on their antiviral activities [32–38], and individual ISGs, including immunity-related GTPases and GBPs, are known to localize to microbial vacuoles and to restrict bacterial infection [39–44]. However, the function of IFNs and ISGs during bacterial infections have not been systematically examined, and the molecular mechanism of IFN-mediated restriction of many bacterial infections remains unknown.

**Fig 9:**
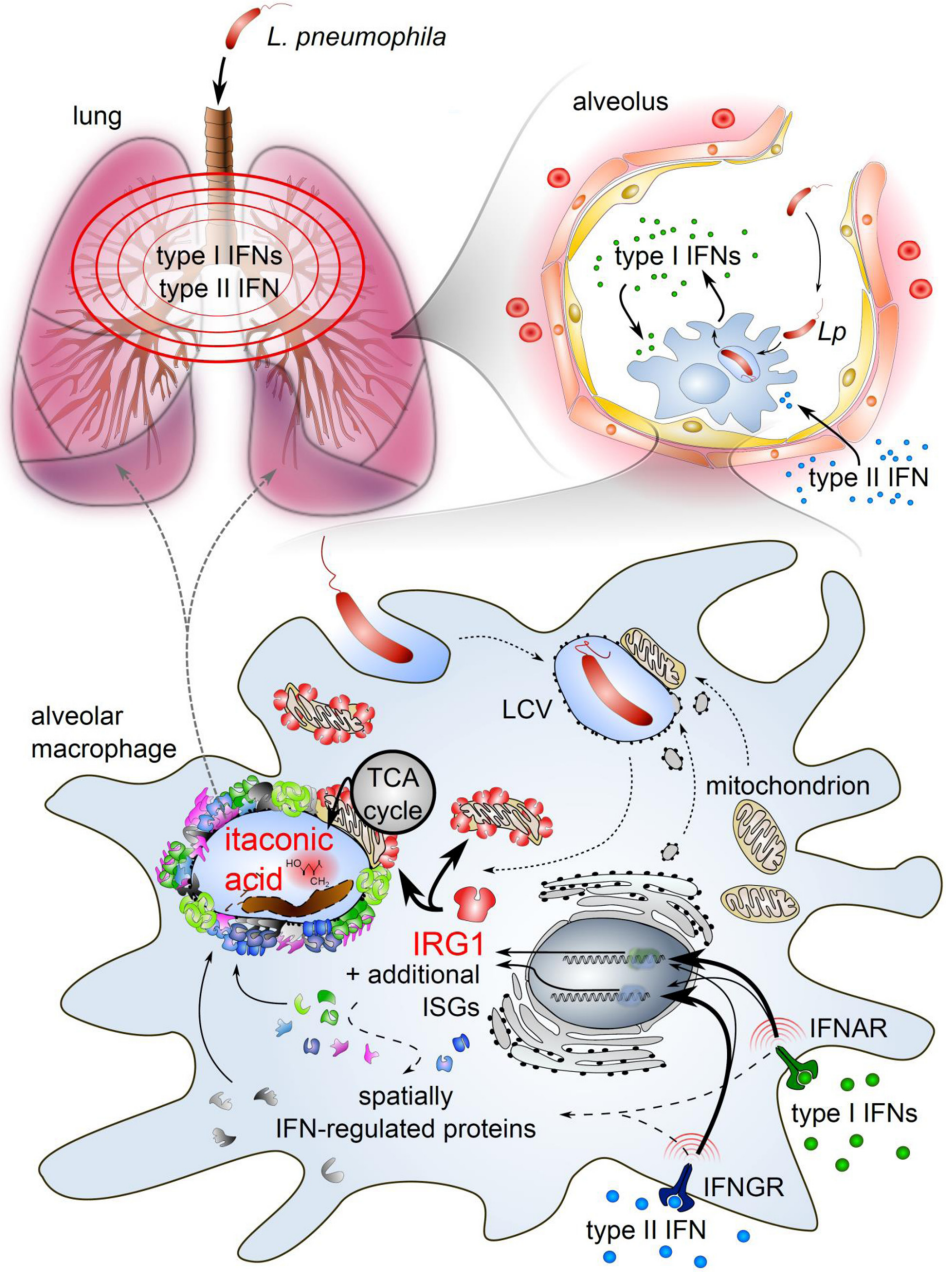
Overview of the type I and II IFN-driven, alveolar macrophage-intrinsic defense pathway that restricts *L*. *pneumophila* during lung infection (as discussed in the text).

We therefore globally profiled the effects of type I and II IFNs on the transcriptome and subcellular proteome during *L*. *pneumophila* infection, thereby providing an important resource for IFN-mediated effects on basic cellular functions during infection. We demonstrate that both IFNs are master regulators of gene expression. Within macrophages, IFNs induce extensive remodeling of bacterial vacuoles, thereby altering their permissiveness for bacterial growth (Fig 9). Neither types of IFNs disturbed the establishment of the LCV but targeted several proteins involved in nucleic acid detection, antigen presentation and antibacterial defense to bacterial vacuoles. Furthermore, we demonstrate that IFN-dependent activation of CD11c^+^ cells (most likely alveolar macrophages) is critical for restricting infection *in vivo*.

Interestingly, the comparison of our proteomic data with the INTERFEROME database as well as our transcriptomic data indicates that a large subset of IFN-dependently LCV-enriched proteins is not transcriptionally regulated. This reveals a hitherto unknown mode of action of IFNs, and suggests that IFNs are able to control the spatial distribution of a subset of proteins, potentially via the activation of signaling molecules that control protein recruitment.

Mitochondrial proteins account for almost one third of identified LCV proteins, whereas the previously reported proteome of latex bead-phagosomes contained only 3–4% mitochondrial proteins [45]. Mitochondria have long been known to attach to LCVs as well as to other microbe-containing vacuoles [6,46,47]. The mechanisms underlying mitochondrial attachment, and most importantly, their biological function at the LCV remain, however, largely unknown. *L*. *pneumophila* secretes a mitochondrial carrier protein (LncP) through its T4SS [48] and might thereby actively recruit the organelles as a source of energy or nutritional metabolites. Alternatively, mitochondria are actively recruited to phagosomes that contain bacteria by a Toll-like receptor (TLR)-dependent mechanism [49]. The TLR-mediated recruitment of mitochondria and the concomitant IFN-dependent up-regulation of antimicrobial proteins within this organelle might thus represent a combined strategy of the immune system to counteract intravacuolar pathogens.

We demonstrate for the first time that the mitochondrial protein IRG1 localizes in close association with a microbial vacuole, and indicate that IRG1 activity is able to restrict *Legionella* inside their LCVs. As discussed above, this IRG1 accumulation on LCVs is most likely dependent on IFN-mediated upregulation of IRG1 within mitochondria and *Legionella*- and/or TLR-dependent recruitment of those organelles to LCVs.

IRG1 has recently been identified as an enzyme catalysing the production of itaconic acid following decarboxylation of the tricarboxylic acid cycle metabolite cis-aconitate [30]. Itaconic acid was found to exert bacteriostatic effects on *Mycobacteria* and *Salmonella* in liquid bacterial cultures [30]. We demonstrate that itaconic acid production *in vivo* is entirely dependent on IFN signals. In addition, we report for the first time a directly bactericidal effect of itaconic acid on different bacterial pathogens, which contrasts to the merely bacteriostatic activity recently reported [30]. Such different effects might be reflective of metabolic differences amongst these bacterial species. In *M*. *tuberculosis*, itaconic acid is thought to inhibit bacterial growth by inhibiting the glyoxylate shunt [30,50]. However, this pathway is believed to be absent in *L*. *pneumophila* [51]. We speculate that the bactericidal activity of itaconic acid on *L*. *pneumophila* involves the accumulation of toxic propionyl-CoA concentrations following inhibition of isocitrate lyase or methylisocitrate lyase [30,52], or the blocking of other pathways. We further assume that the IRG1/itaconic acid pathway acts in concert with established antibacterial factors such as IRGM1 and GBPs to completely eliminate *L*. *pneumophila* in IFN-activated macrophages and mice [15,53].

Type II IFN is well-known for its activation of antibacterial immunity to most intravacuolar bacteria, whereas type I IFNs have been shown to either enhance or inhibit those responses [54]. Using an unbiased, quantitative approach we show here that, in principle, both types of IFNs are able to induce most known antibacterial ISGs (e.g. GBPs, immunity-regulated GTPases, IRG1). One could speculate that the differential roles of type I IFNs in various bacterial infections might be explained by differences in the architecture of the bacterial vacuoles, and the relative contribution of IFN-dependent defense systems versus other intracellular or extracellular immune mechanisms.

In conclusion, our study provides for the first time comprehensive insight into the transcriptional and spatial regulations induced by type I and II IFNs that lead to critical modifications in the proteome of bacterial vacuoles, and it identifies a novel IFN-controlled defense pathway against *L*. *pneumophila* infection. In the future, therapeutic stimulation of IRG1 or delivery of encapsulated itaconic acid might be useful to fight intracellular and multidrug-resistant bacteria.

## Materials and Methods

### Bacteria

The *L*. *pneumophila* serogroup 1 strain JR32, the Δ*flaA* mutant, JR32 expressing eGFP or DsRed and the culture conditions have been described previously [11,55]. Δ*flaA* mutants expressing eGFP or DsRed have been generated in this study using plasmids published recently [55]. A clinical isolate of methicillin resistant *Staphylococcus aureus* (MRSA) has been obtained from the Charité microbiology department, the multidrug-resistant *Acinetobacter baumannii* isolate A9703 was kindly provided by Harald Seifert, University Cologne, Germany. *S*. *aureaus* and *A*. *baumannii* were cultured on LB agar.

### Ethics statement

All animal experiments were approved by institutional (Charité –Universitätsmedizin Berlin) and governmental animal welfare committees (LAGeSo Berlin; approval IDs G0446/08, G0278/11, G0440/12).

### Mice

*Casp11* ^*-/-*^, *Ifnar* ^*-/-*^, *Ifngr* ^*-/-*^, *Rip3* ^*-/-*^, *Ifnar/Ifngr* ^-/-^ and *Nos2* ^*-/-*^ mice were on a C57BL/6 background [15,56,57]. C57BL/6 CD45.1 mice and transgenic CD11c-DTR-GFP [58] mice were bred and maintained at the University of Melbourne.

### *In vivo* infection

All mice used were on C57BL/6J background, 8–10 weeks old and female. Anaesthetized mice were intranasally infected with 1 × 10^6^ *L*. *pneumophila* in 40 μl of PBS [15].

### RNA extraction, microarray analysis and upstream regulator analysis

Lungs were flushed via the pulmonary artery with sterile saline and homogenized in Trizol (Life Technologies) [59]. Homogenized lungs were pooled (5 mice per group) and RNA extraction was carried out according to manufacturer’s instructions. RNA amounts were estimated with a NanoDrop 1000 UV-Vis spectrophotometer (Kisker) and RNA integrity was confirmed using an Agilent 2100 Bioanalyzer with a RNA Nano 6000 microfluidics kit (Agilent Technologies). Microarray analysis was performed as dual-color hybridizations. In order to compensate dye-specific effects and to ensure statistically relevant data, color-swap dye-reversal hybridizations were performed [60]. RNA labeling was done with a two-color Quick Amp Labeling Kit according the supplier’s recommendations (Agilent Technologies). In brief, mRNA was reverse transcribed and amplified using an oligo-dT-T7 promoter primer, and labeled with Cyanine 3-CTP or Cyanine 5-CTP. After precipitation, purification, and quantification, 1.25 μg of each labeled cRNA was fragmented and hybridized to whole mouse genome 4x44K multipack microarrays (Design ID 014868) according to the manufacturer’s protocol (Agilent Technologies). Scanning of microarrays was performed with 5 μm resolution using a G2565CA high-resolution laser microarray scanner (Agilent Technologies) with XDR extended range. Microarray image data were analyzed and extracted with the Image Analysis/Feature Extraction software G2567AA v. A.10.10.1.1 (Agilent Technologies) using default settings and the protocol GE2_1010_Sep10. The extracted MAGE-ML files were subsequently analyzed with the Rosetta Resolver, Build 7.2.2 SP1.31 (Rosetta Biosoftware). Ratio profiles comprising single hybridizations were combined in an error-weighted fashion to create ratio experiments. A 1.5-fold change expression cut-off for ratio experiments was applied together with anti-correlation of ratio profiles, rendering the microarray analysis highly significant (p < 0.01), robust, and reproducible. The data discussed in this publication have been deposited in NCBI's Gene Expression Omnibus and are accessible through GEO Series accession number GSE60085. Genes identified to be significantly up-regulated upon *L*. *pneumophila* infection (> 2-fold increase, p < 0.05 in infected versus PBS treated WT mice; S3 Dataset) were analyzed for their predicted upstream regulators using the Ingenuity Pathway Analysis (IPA) software (Ingenuity System). Only upstream regulators with an activation z-score > 2 (predicted activators) were considered and further categorized in respective groups.

### Bone marrow chimeric mice

Chimeras were generated as described recently [61] (Fig 2A). Briefly, CD45.1^+^ mice were lethally irradiated twice with 550 cGy and reconstituted with a 1:1 mix of 1.5 × 10^6^ bone marrow cells from C57BL/6 WT or *Ifnar/Ifngr* ^-/-^ and transgenic CD11c-DTR-GFP mice (all CD45.2^+^). Chimeric mice were allowed to reconstitute for at least 10 weeks. Only those mice that contained < 10% host cells were included in experiments. Depletion of CD11c^+^ cells was achieved by injection of CD11c-DTR-GFP chimeric mice intraperitoneally three times with 100 ng diphtheria toxin (Sigma-Aldrich) on days -2, +1 and +4 prior to and during infection.

### Flow cytometry

Preparation of lung cells for the determination of cell exchange in chimeric mice has been described [62]. The lung cell suspension was labelled with anti-panCD45 (30-F11, eBioscience), anti-CD45.1 (A20, BD Pharmingen), anti-CD45.2 (104, BD Pharmingen), anti-Ly6G (1A8, BD Pharmingen), anti-CD11c (N418, eBioscience) anti-MHC-II (M5/114.15.2, eBioscience), anti-Siglec-F, (E50-2440, BD Pharmingen) and anti-CD64 (X54-5/7.1, BD Pharmingen). Cells were analyzed on a Becton Dickinson LSRFortessa flow cytometer using FACSDIVA software (BD Biosciences).

### Cell isolation

Bone marrow-derived macrophages (BMMs) were prepared from femurs and tibiae. Alveolar macrophages (AMs) were isolated by extensive bronchoalveolar lavage and purity was checked by flow cytometry.

### Cell transfection and infection

BMMs and AMs were transfected with control non-silencing or a mix of two gene-specific siRNAs (S1 Table) 24 h prior to infection (Life Technologies) using HiPerfect (Qiagen), and with EGFP (pEGFP-N1, Clontech) or full-length murine IRG1 (NM_008392) with a carboxy-terminal TurboGFP (pCMV6-AC-GFP, OriGene) or Myc-DDK (pCMV6-Entry, OriGene) tag 48 h prior to infection using ViaFect (Promega). BMMs and AMs were infected with *L*. *pneumophila* wt or Δ*flaA*, centrifuged at 200 *g* for 5 min and incubated for the indicated time intervals. Where indicated, cells were incubated either with IFNβ, IFNγ or both 16–18 h prior to and during infection at a concentration of 50 U/ml unless stated otherwise. For intracellular replication assays, cell death and mitochondrial ROS assays, BMMs or AMs were infected with *L*. *pneumophila*, centrifuged at 200 *g* for 5 min and incubated at 37°C for 30 min. Cells were washed with PBS and were further incubated with RPMI with 15% L cell supernatant and 10% FCS (BMMs) or 10% FCS (AMs) containing 50 μg/ml gentamicin for 1 h in order to kill extracellular bacteria.

### Quantitative RT-PCR

Total RNA was isolated from BMMs or lung homogenates using the PerfectPure RNA purification system (5 Prime) or Trizol (Life Technologies), respectively, reverse-transcribed using high capacity reverse transcription kit (Applied Biosystems), and quantitative PCR was performed using TaqMan assays (Life Technologies) or self-designed primer sets, respectively (S2 Table), on an ABI 7300 instrument. The input was normalized to the average expression of GAPDH and relative expression (relative quantity, RQ) of the respective gene in untreated cells or PBS-treated mice was set as 1.

### Cell death and mitochondrial ROS measurement

Cells were detached using ice cold PBS containing 2 mM EDTA and stained for mitochondrial ROS (MitoSOX Red, Life Technologies) or cell death (7-AAD, eBioscience or LIVE/DEAD fixable red dead cell stain, Life Technologies). Proportions of mitochondrial ROS producing (MitoSOX^+^) or dead (7AAD^+^ or LIVE/DEAD^+^) cells were determined in infected (GFP^+^) and uninfected (GFP^-^) cell populations by flow cytometry (FACScan, BD or MACSQuant, Miltenyi Biotec). Data analysis was done using FlowJo software (Tree Star).

### Immunoblotting

For immunoblotting cells were lysed in SDS- and 1% NP40-containing lysis buffer, cleared extracts were separated by SDS-PAGE and SDS-gels were blotted onto Hybond nitrocellulose membranes. Antibodies against IRG1 (HPA040143, Sigma-Aldrich) and actin (sc-1616, Santa Cruz) followed by respective fluorophore-linked secondary antibodies (Rockland) were used and blots analyzed using an Odyssey infrared imaging system (Li-Cor).

### Confocal laser scanning, structured illumination and time-lapse microscopy

BMMs were seeded onto glass coverslips or high precision glass coverslips for structured illumination microscopy. Where indicated, cells were stained with MitoTracker Orange (Life Technologies) approximately 2 h prior infection. Cells were fixed with 3% PFA or aceton/methanol if MitoTracker was used. For homogenization, cells were seeded in 6 well plates, infected and 2 h p.i. washed with PBS, scraped in homogenization buffer (20 mM Hepes, 250 mM sucrose, 0.5 mM EGTA, pH 7.2) and homogenized using a ball homogenizer (Isobiotec). Homogenates were centrifuged onto glass coverslips and fixed with 3% PFA. For intracellular staining, cells were permeabilized with 0.1% triton x-100 and blocked with 5% FCS. Where indicated cells were stained with an affinity purified rabbit anti-SidC [63] and a mouse anti-*Legionella pneumophila* (ab69239, Abcam) antibody followed by the respective anti-rabbit Alexa Fluor 633-conjugated (Molecular Probes) and anti-mouse DyLight 405-conjugated (Thermo Scientific) secondary antibody. Samples were mounted on slides using PermaFluor (Thermo Scientific) containing DAPI or Mowiol (Sigma-Aldrich). For confocal laser scanning microscopy samples were examined using a LSM 780 microscope (objective: Plan Apochromat 63×/1.40 oil DIC M27, Carl-Zeiss). Structured illumination microscopy was performed on an ELYRA PS.1 microscope (objective: Plan Apochromat 63×/1.40 oil DIC M27, Carl-Zeiss). Data sets were acquired with five grating phases and three rotations, post-processed in ZEN (Carl-Zeiss) using automatically determined parameters, and colour channels were subsequently aligned based on parameters determined from control measurements with multispectral beads performed with identical instrument settings. For time-lapse confocal microscopy cells were seeded in 8 well μ-slides (Ibidi) and stained with MitoTracker Orange (Life Technologies) approximately 2 h prior infection. Images were acquired on a LSM 780 microscope (objective: Plan Apochromat 63×/1.40 oil DIC M27, Carl-Zeiss) at 37°C/5% CO2. All images were processed using ZEN (Carl-Zeiss) and ImageJ software (http://imagej.nih.gov/ij/).

### LCV isolation

BMMs were left untreated or treated with 50 U/ml IFNβ or IFNγ for 16–18 h prior to and during infection and subsequently infected with *L*. *pneumophila* Δ*flaA* for 2 h at a m.o.i of 50. LCVs from BMMs were isolated as described previously for RAW264.7 cells and amoeba [27,64]. Briefly, cells were washed with PBS, scraped in homogenization buffer (20 mM Hepes, 250 mM sucrose, 0.5 mM EGTA, pH 7.2), homogenized using a ball homogenizer (Isobiotec), and incubated with an anti-SidC antibody [63] followed by a secondary anti-rabbit antibody coupled to magnetic beads (Miltenyi Biotec). The LCVs were separated in a magnetic field and further purified by a Histodenz density gradient centrifugation step.

### Proteomic analysis

Isolated LCV from 4 IFNβ, 5 IFNγ, and 6 untreated biological replicates were analyzed. LCV samples were solubilized in 1% RapiGest (Waters) in 50 mM Tris pH 8.0, reduced with 10 mM tris (2-carboxyethyl)phosphine (TCEP) (Pierce), and heated at 70°C for 10 min. After cooling, proteins were alkylated in 10 mM iodoacetamide (Sigma-Aldrich), and alkylation was quenched in 20 mM DTT. Protein concentrations were measured by the EZQ assay (Life Technologies), and 8 μg of protein was digested by trypsin overnight at 30°C, after diluting the Rapigest concentration to 0.1%. Rapigest was removed from the sample by acidification to 2% trifluoroacetic acid (TFA) and incubation at 37°C for 1 h, followed by centrifugation at 14k *g* for 30 min. Peptides were then desalted with Microspin C18 solid phase extraction columns (The Nest Group). After drying down, peptides were redissolved in 1% TFA.

For each sample, 2 μg of peptides were analyzed on an Orbitrap Velos Pro mass spectrometer coupled to an Ultimate 3000 UHPLC system with a 50 cm EasySpray analytical column (75 μm ID, 3 μm C18) in conjunction with a Pepmap trapping column (100 μm x 2 cm, 5 μm C18) (Thermo-Fisher Scientific). Acquisition settings were: lockmass of 445.120024, MS1 with 60,000 resolution, top 20 CID MS/MS using Rapid Scan, monoisotopic precursor selection, unassigned charge states and z = 1 rejected, dynamic exclusion of 60s with repeat count 1. 6 h linear gradients were performed from 3% solvent B to 35% solvent B (solvent A: 0.1% formic acid, solvent B: 80% acetonitrile 0.08% formic acid) with a 30 min washing and re-equilibration step [65].

Protein identification and quantification were performed using MaxQuant Version 1.4.1.2 [66] with the following parameters: stable modification carbamidomethyl (C); variable modifications of methionine oxidation, and protein N-terminal acetylation, and 2 missed cleavages. Searches were conducted using a Uniprot-Trembl *Mus musculus* database downloaded May 1, 2013, *Legionella pneumophila* strain Philadelphia 1 downloaded December 4, 2013, and common contaminants. Identifications were filtered at a 1% false-discovery rate (FDR) at the protein level, accepting a minimum peptide length of 7. Quantification used only razor and unique peptides, and required a minimum ratio count of 2. “Re-quantify” and “match between runs” were enabled.

### Gene Ontology (GO) analysis

The host proteins identified in all six LCV samples from untreated macrophages (S1 Dataset) were analyzed for overrepresented cellular components using g:Profiler (http://biit.cs.ut.ee/gprofiler/) [67] with default settings including g:SCS algorithm for multiple testing correction. All overrepresented child terms of the GO term *intracellular membrane-bounded organelle* (GO:0043231, p = 3.55 × 10^−300^) were extracted. The total result of GO enrichment analysis for cellular components (CC) can be found in S1 Dataset. To identify and visualize biological processes that are overrepresented at LCVs of untreated cells, the same list of proteins was analyzed with BiNGO [68] for Cytoscape [69] using default settings including hypergeometric testing and Benjamini & Hochberg FDR correction. Significance level cut-off was set to < 10^−10^ (terms with p-values > 10^−10^ are depicted if p-value of final child term was < 10^−10^). The total result of GO enrichment analysis for biological process (BP) can be found in S1 Dataset.

### Integrated STRING network analysis

Proteins identified in IFN-treated (in 4 of 4 IFNβ or 5 of 5 IFNγ treated samples) but not in untreated samples (≤ 1 of 6 samples; hereafter called “qualitative changers”) and proteins with significant higher abundance in IFN-treated versus untreated samples (log_2_ LFQ intensity ratio ≥ 1, p < 0.05; hereafter called “quantitative changers”) (S2 Dataset) were combined and analyzed for protein-protein interaction networks using STRING database (http://string-db.org/). The identified network was extracted and loaded into Cytoscape [69] for visualization; only interactions with a minimum STRING combined score of 0.400, which represents the default medium confidence level in STRING, were kept. For identification of subnetworks of overrepresented biological functions, the combined protein list was analyzed by g:Profiler (http://biit.cs.ut.ee/gprofiler/) [67]. Protein lists of overrepresented GO terms were extracted and subnetworks were built using STRING and Cytoscape. To identify proteins within the networks that were also transcriptionally induced by IFNs upon *in vivo L*. *pneumophila* infection, the combined list of qualitative and quantitative changing proteins was compared to genes with a >2-fold change (p < 0.05) in *L*. *pneumophila* infected *Ifnar*/*Ifngr* ^-/-^ versus WT mice (S3 Dataset). To cross-reference gene names from transcriptome analysis and Uniprot identifier from proteome analysis, both lists were uploaded to STRING and respective output lists were compared against each other. For identification of ISGs the protein list was also compared against the INTERFEROME database [28].

### Gas chromatography-mass spectrometry (GC-MS) analysis of itaconic acid production

10^6^ BMMs per well were left untreated, were incubated either with 50 U/ml IFNβ or IFNγ for 16–18 h or were infected with *L*. *pneumophila* for 24 h. Where indicated cells were transfected with control non-silencing or a mix of two gene-specific siRNAs as described above 24 h prior to infection. After washing with PBS, metabolism was stopped adding 200 μl cooled 50% MeOH (-20°C) and cells were collected by scraping in the MeOH solution. Cells from 6 wells were pooled, 240 μl chloroform were added, samples centrifuged for 10 min at 10k g and supernatant containing polar metabolites was dried under vacuum overnight. For *in vivo* experiments mice were infected with *L*. *pneumophila* wt or left untreated. 2 d p.i. lungs were flushed with sterile PBS, shock frozen in liquid nitrogen and stored at -80°C. Lung tissue was homogenized using a Precellys24 bead homogenizer in chloroform (6 mL/g), methanol (6 mL/g), and distilled water (4 mL/g). Samples were centrifuged for 10 min at 10k g and supernatant containing polar metabolites was dried under vacuum overnight. For GC/MS analysis samples were processed using protocols and machine settings described elsewhere [70]. Data were analyzed using ChromaTOF (Leco) and the custom software MetMax [71]. Data were normalized on mean of total area of all analyzed metabolites (*in vitro* samples) or on internal standard (*in vivo* samples) and average amount of itaconic acid in untreated cells or control mice was set as 1.

### Itaconic acid growth inhibition and killing assay

For growth inhibition bacteria were grown in AYE broth containing indicated amounts of itaconic acid. OD_600_ was determined over time. For killing assays bacteria were resuspended in PBS and respective amounts of itaconic acid, acetic acid or citric acid were added. Bacteria were incubated at 37°C and plated at indicated time points to assess viability.

### Statistical analysis

Data were statistically analyzed using GraphPad Prism software. Groups were compared with two-tailed Mann-Whitney U test or, for multiple-group comparisons with Kruskal-Wallis analysis of variance followed by Dunn’s multiple comparison test. Differences with p < 0.05 were considered statistically significant.

### Accession code

GEO: GSE60085.

## Supporting Information

S1 Fig Proportions of CD11c^+^ GFP^+^ (undepleted) cells in DTX treated chimeric mice. CD45.1 recipient mice were lethally irradiated and repopulated with a 1:1 mixture of bone-marrow cells from CD45.2 transgenic CD11c-DTR-GFP and *Ifnar/Ifngr* ^-/-^ or WT donor mice. (A) Repopulation with CD45.2 donor cells within CD45.1 recipient mice was assessed by flow cytometry of whole lung cells (representative dot plot). (B) Cell proportions were determined in total lung homogenates from CD11c-DTR-GFP / WT + DTX and CD11c-DTR-GFP / *Ifnar*/*Ifngr* ^-/-^ + DTX mice by flow cytometry and gating on CD45^+^ CD11c^+^ (all CD11c^+^ cells), CD45^+^ CD11c^+^ CD64^+^ / SiglecF^+^ (macrophages / monocytes) or CD45^+^ CD11c^+^ CD64^-^ SiglecF^-^ MHC-II^hi^ (dendritic cells). Only mice with <10% GFP^+^ (of all CD11c^+^) cells were considered for analysis depicted in Fig 2C. No cut-off was applied for analysis depicted in Fig 2B.
(PDF)

S2 Fig Macrophage activation by IFNs restricts intracellular growth of *L*. *pneumophila*. (A-C) Intracellular growth of *L*. *pneumophila* Δ*flaA* in WT BMMs left untreated or treated with IFNβ, IFNγ or both 16–18 h prior to and during infection. (D) Intracellular growth of *L*. *pneumophila* wt and Δ*flaA* in WT and *Ifnar* ^-/-^ BMMs. Data represent mean + s.e.m. of 2 (B), 4 (C) or 5 (A, D) experiments done in triplicates. * p<0.05, ** p<0.01, *** p<0.001, no indication if not significant (two-tailed Mann-Whitey U test), significance was tested against untreated control (A-C) or between wild-type and knock-out cells for each condition (D).
(PDF)

S3 Fig *L*. *pneumophila* restriction in macrophages is largely independent of IFN-driven cell death. Cell death of infected (GFP^+^) cells (A; gating strategy) in WT BMMs left untreated or treated with 50 U/ml IFNβ or IFNγ 16–18 h prior to and during infection (D), *Ifnar* ^-/-^ (B, C), and *Rip3* ^-/-^ (E) BMMs infected with *L*. *pneumophila* wt or Δ*flaA* expressing eGFP was determined by flow cytometry. Data represent mean + s.e.m. of 2 (E) or 4 (C, D) experiments done in triplicates. * p<0.05, ** p<0.01, no indication if not significant (two-tailed Mann-Whitey U test), significance was tested against untreated control (D) or between wild-type and knock-out cells for each condition (C, E). (B) Representative blots of 4 independent experiments done in triplicates (summarized in C) are shown.
(PDF)

S4 Fig *L*. *pneumophila* restriction in macrophages is largely independent of RIP3, caspase-11 and iNOS. Intracellular growth of *L*. *pneumophila* wt and Δ*flaA* in WT, *Rip3* ^-/-^ (A, D), *Casp11* ^-/-^ (B, E) and Nos2^-/-^ (C, F) BMMs left untreated (D-F) or treated with IFNβ or IFNγ 16–18 h prior and during infection (A-C). Data represent mean + s.e.m. of 2 (A, B, D, E) or 3 (C, F) experiments done in triplicates. No significant differences between wild-type and knock-out cells were found for any condition (two-tailed Mann-Whitey U test).
(PDF)

S5 Fig Proteins involved in metabolic processes and transport and localization are strongly enriched at LCVs. GO enrichment analysis for biological processes (BP) of the 2307 host proteins identified in untreated LCV samples using BiNGO (Cytoscape). Hierarchical structure, read from inside (first node, blue encircled) to outside (final nodes, pink encircled). Subnetworks of highly enriched biological processes are highlighted (metabolic process, transport/localization, biological regulation, immune system process). Significance cut-off value for visualization was set to 10^−10^, ancestor terms with p > 10^−10^ are depicted if final child term had p-value < 10^−10^. Tabular outline of whole analysis including exact p-values and full lists of proteins for each GO term can be found in S2 Dataset.
(PDF)

S6 Fig Proteins differentially targeted to the LCV upon IFNβ or IFNγ treatment. Quantitative proteomic analysis of LCVs isolated 2 h p.i. with *L*. *pneumophila* Δ*flaA* from BMMs left untreated or treated with 50 U/ml IFNβ or IFNγ 16–18 h prior to and during infection. Bar graphs show top 20 proteins with a significant higher (red) or lower (green) abundance at LCVs from IFNβ- (A, B) or IFNγ- (D, E) treated BMMs compared to untreated cells, and direct comparison of IFNβ- versus IFNγ-treated samples (C, F). Bar graphs correspond with volcano blots depicted in Fig 3C–3E. Proteomic analysis was done from 6 (untreated), 5 (IFNγ) and 4 (IFNβ) individual LCV isolations.
(PDF)

S7 Fig IRG1 is transcriptionally regulated by type I and type II IFNs. *Irg1* gene expression in WT BMMs left untreated or treated with 50 U/ml IFNβ or IFNγ for 16–18 h was determined by qRT-PCR. Data are mean + s.e.m. of 2 independent experiments done in triplicates.
(PDF)

S8 Fig Mitochondria stay in close proximity to intracellular *L*. *pneumophila*. Representative frames from time-lapse confocal imaging of mitotracker stained (red) WT BMMs infected with *L*. *pneumophila* Δ*flaA* expressing eGFP (green). Imaging starts approximately 2 h p.i.. White arrow points toward a single mitochondrion staying in close proximity of the intracellular *L*. *pneumophila*, while other mitochondria move dynamically within the cell (open white arrowhead). The full sequence of frames with a 1-minute-time resolution can be found in S1 Video.
(PDF)

S9 Fig Mitochondrial ROS production in *L*. *pneumophila*-infected cells is largely independent of type I IFN signalling and IRG1. WT BMMs transfected with siRNAs 24 h prior to infection (A), or WT and *Ifnar* ^-/-^ BMMs (B) were infected with eGFP-expressing *L*. *pneumophila* wt and proportions of mitochondrial ROS producing (MitoSOX^+^) cells were determined by flow cytometry in infected (GFP^+^) and uninfected (GFP^-^) populations, respectively. Data represent mean + s.e.m. of 2 (B) or 4 (A) experiments done in triplicates.
(PDF)

S1 Dataset *In vivo* transcriptome data of *L*. *pneumophila*-infected wild-type and IFN-receptor knock-out mice. Tabular outline of all genes significantly up- or down-regulated in WT mice upon *L*. *pneumophila* infection and their regulation in *Ifnar* ^-/-^, *Ifngr* ^-/-^ and *Ifnar*/*Ifngr* ^-/-^ mice upon infection.
(XLSX)

S2 Dataset LCV proteome data of *L*. *pneumophila*-infected untreated BMMs. All host- and *Legionella*-derived proteins identified in samples of LCVs isolated from *L*. *pneumophila*-infected resting BMMs are listed. Tabular outline of total results of GO analyses of all identified host proteins for cellular component (CC; as depicted in Fig 3B), and biological process (BP; as depicted in S5 Fig) computed using G-profiler and BiNGO for Cytoscape, respectively.
(XLSX)

S3 Dataset LCV proteome data untreated compared to IFN-treated BMMs. Quantification of host-derived proteins identified in samples of LCVs from resting BMMs against LCVs from IFN-treated BMMs as well as quantification of proteins from LCVs of IFNβ against IFNγ treated BMMs.
(XLSX)

S1 Video Mitochondria stay in close proximity to intracellular *L*. *pneumophila*. Time-lapse confocal imaging of mitotracker stained (red) WT BMMs infected with *L*. *pneumophila* Δ*flaA* expressing eGFP (green). Imaging starts approximately 2 h p.i. and single frames were taken every minute. See also S8 Fig.
(AVI)

S1 Table Oligonucleotides used for RNAi in this study. (DOCX)

S2 Table TaqMan assays and primer sets used for quantitative real-time PCR in this study. (DOCX)
