# Supplementary figures and images for: IFNs Modify the Proteome of Legionella-Containing Vacuoles and Restrict Infection Via IRG1-Derived Itaconic Acid
Source: PLoS Pathog. 2016 Feb 1;12(2):e1005408. doi: 10.1371/journal.ppat.1005408 (PMC4734697; doi:10.1371/journal.ppat.1005408)

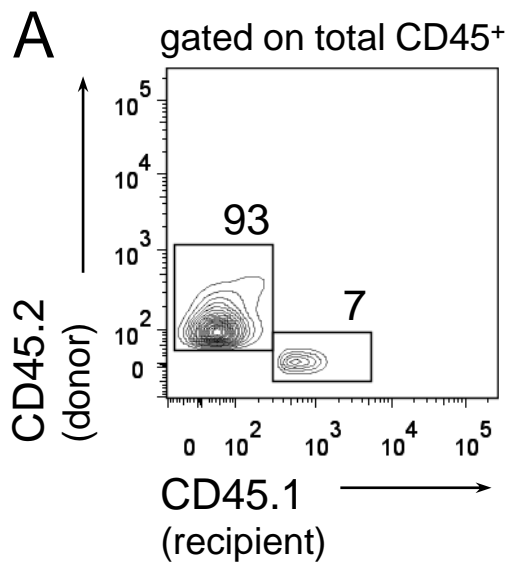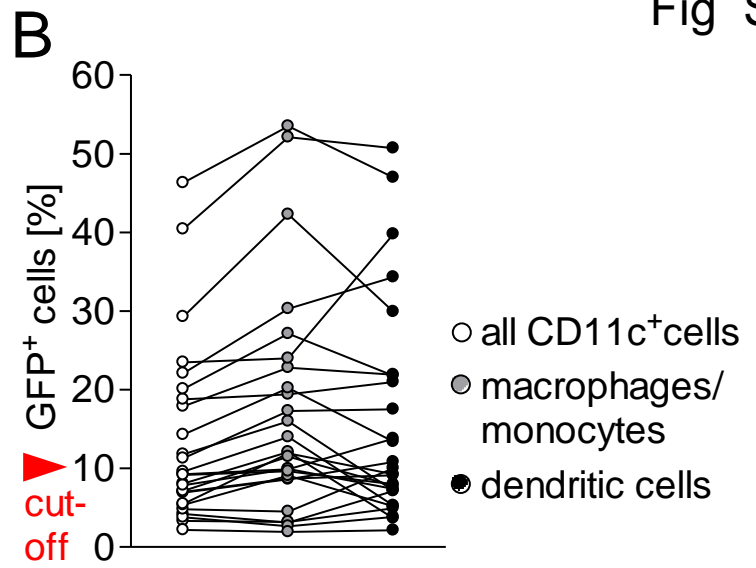

Supplement: S1 Fig — CD45.1 recipient mice were lethally irradiated and repopulated with a 1:1 mixture of bone-marrow cells from CD45.2 transgenic CD11c-DTR-GFP and Ifnar/Ifngr -/- or WT donor mice. (A) Repopulation with CD45.2 donor cells within CD45.1 recipient mice was assessed by flow cytometry of whole lung cells (representative dot plot). (B) Cell proportions were determined in total lung homogenates from CD11c-DTR-GFP / WT + DTX and CD11c-DTR-GFP / Ifnar/Ifngr -/- + DTX mice by flow cytometry and gating on CD45+ CD11c+ (all CD11c+ cells), CD45+ CD11c+ CD64+ / SiglecF+ (macrophages / monocytes) or CD45+ CD11c+ CD64- SiglecF- MHC-IIhi (dendritic cells). Only mice with <10% GFP+ (of all CD11c+) cells were considered for analysis depicted in Fig 2C. No cut-off was applied for analysis depicted in Fig 2B. (PDF) [file ppat.1005408.s001.pdf]

A

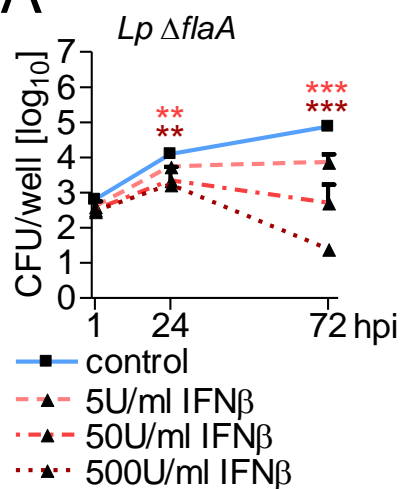

B

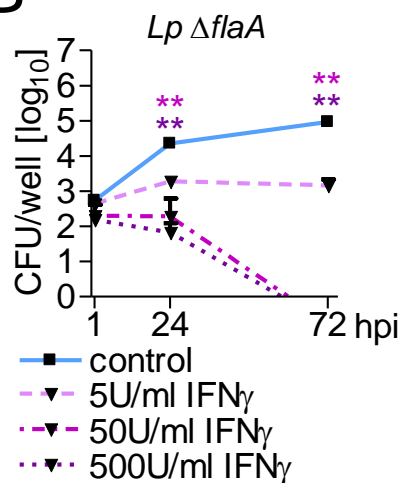

C

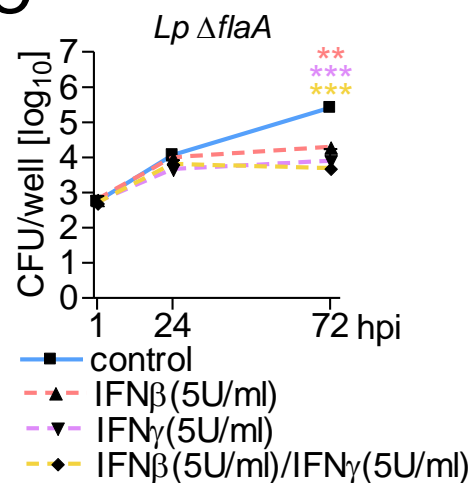

D

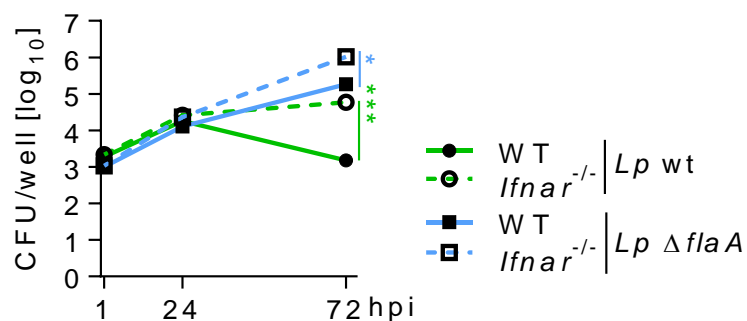

Supplement: S2 Fig — (A-C) Intracellular growth of L. pneumophila ΔflaA in WT BMMs left untreated or treated with IFNβ, IFNγ or both 16–18 h prior to and during infection. (D) Intracellular growth of L. pneumophila wt and ΔflaA in WT and Ifnar -/- BMMs. Data represent mean + s.e.m. of 2 (B), 4 (C) or 5 (A, D) experiments done in triplicates. * p<0.05, ** p<0.01, *** p<0.001, no indication if not significant (two-tailed Mann-Whitey U test), significance was tested against untreated control (A-C) or between wild-type and knock-out cells for each condition (D). (PDF) [file ppat.1005408.s002.pdf]

A

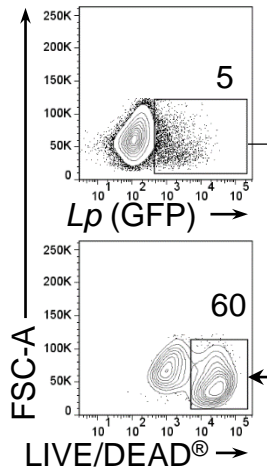

B

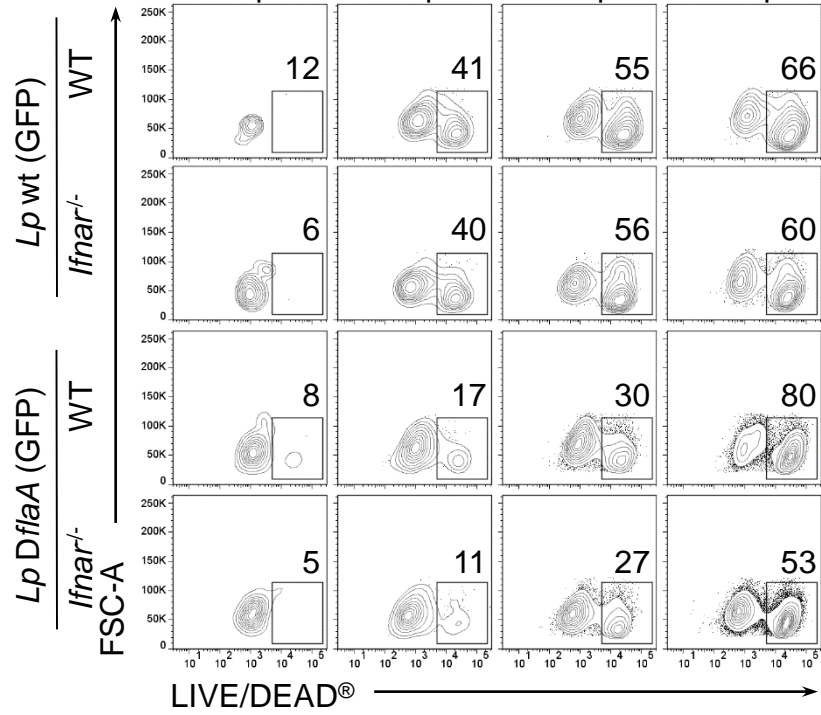

C

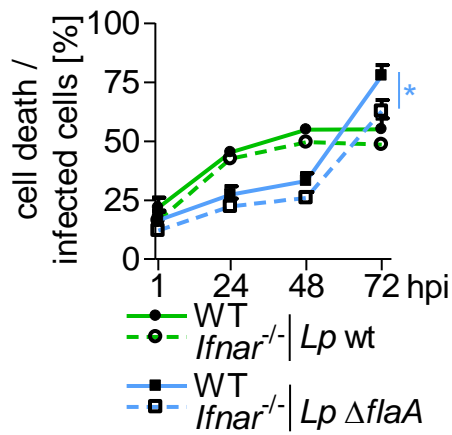

D

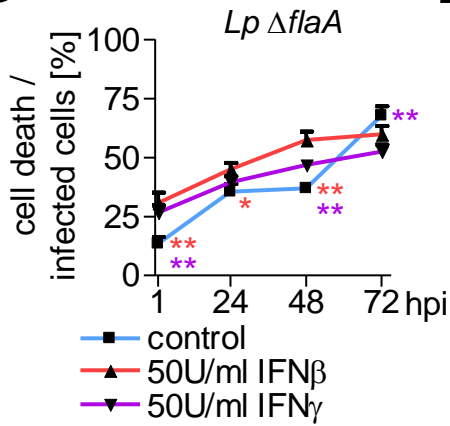

E

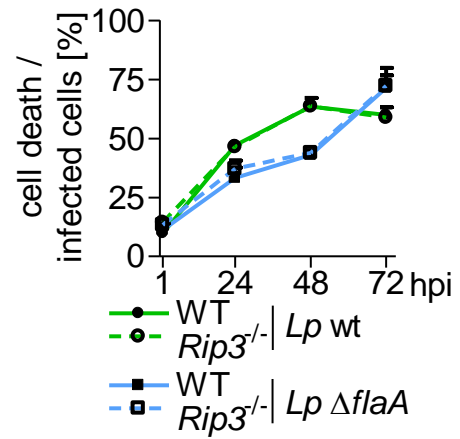

Supplement: S3 Fig — Cell death of infected (GFP+) cells (A; gating strategy) in WT BMMs left untreated or treated with 50 U/ml IFNβ or IFNγ 16–18 h prior to and during infection (D), Ifnar -/- (B, C), and Rip3 -/- (E) BMMs infected with L. pneumophila wt or ΔflaA expressing eGFP was determined by flow cytometry. Data represent mean + s.e.m. of 2 (E) or 4 (C, D) experiments done in triplicates. * p<0.05, ** p<0.01, no indication if not significant (two-tailed Mann-Whitey U test), significance was tested against untreated control (D) or between wild-type and knock-out cells for each condition (C, E). (B) Representative blots of 4 independent experiments done in triplicates (summarized in C) are shown. (PDF) [file ppat.1005408.s003.pdf]

Fig S4

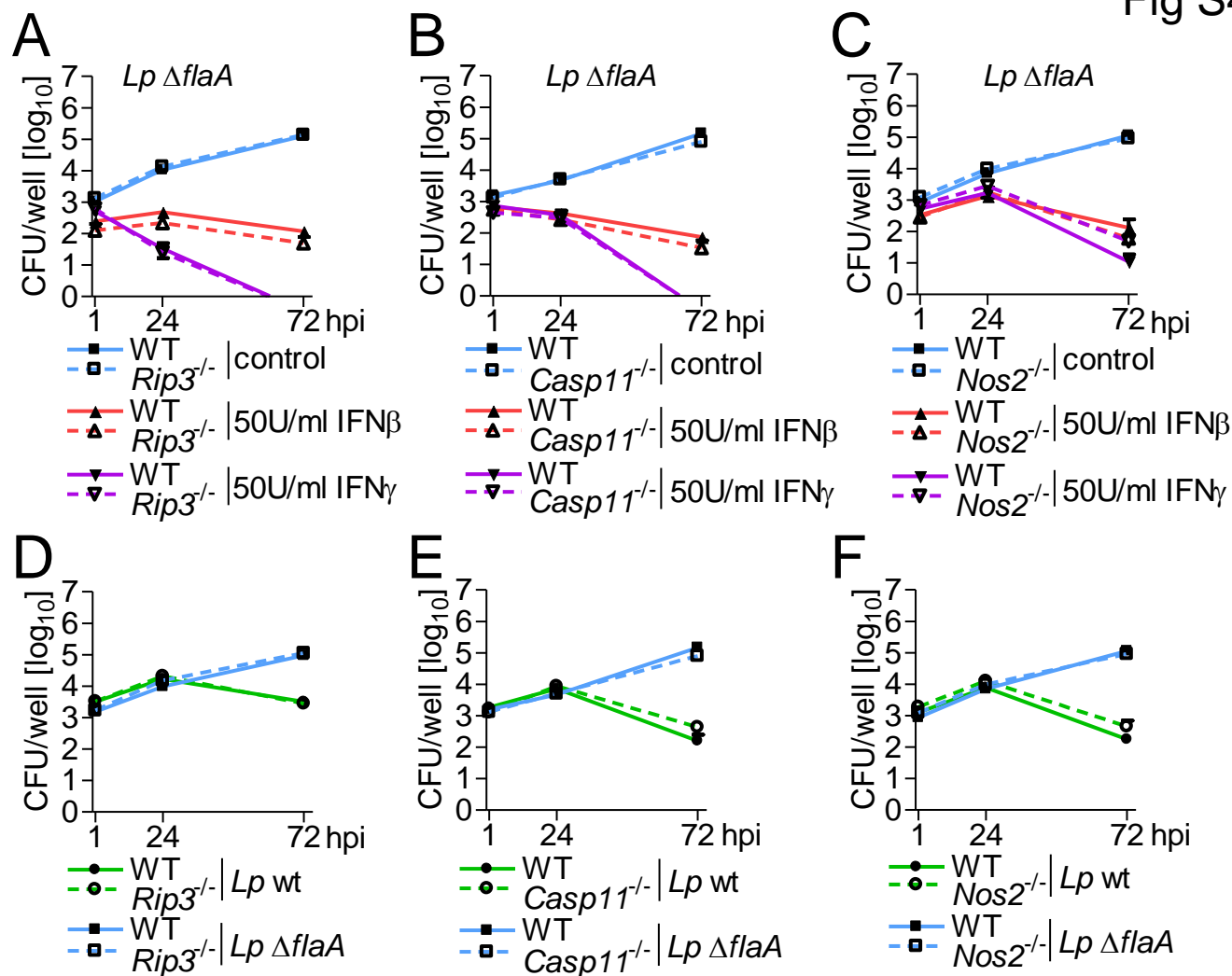

Supplement: S4 Fig — Intracellular growth of L. pneumophila wt and ΔflaA in WT, Rip3 -/- (A, D), Casp11 -/- (B, E) and Nos2-/- (C, F) BMMs left untreated (D-F) or treated with IFNβ or IFNγ 16–18 h prior and during infection (A-C). Data represent mean + s.e.m. of 2 (A, B, D, E) or 3 (C, F) experiments done in triplicates. No significant differences between wild-type and knock-out cells were found for any condition (two-tailed Mann-Whitey U test). (PDF) [file ppat.1005408.s004.pdf]

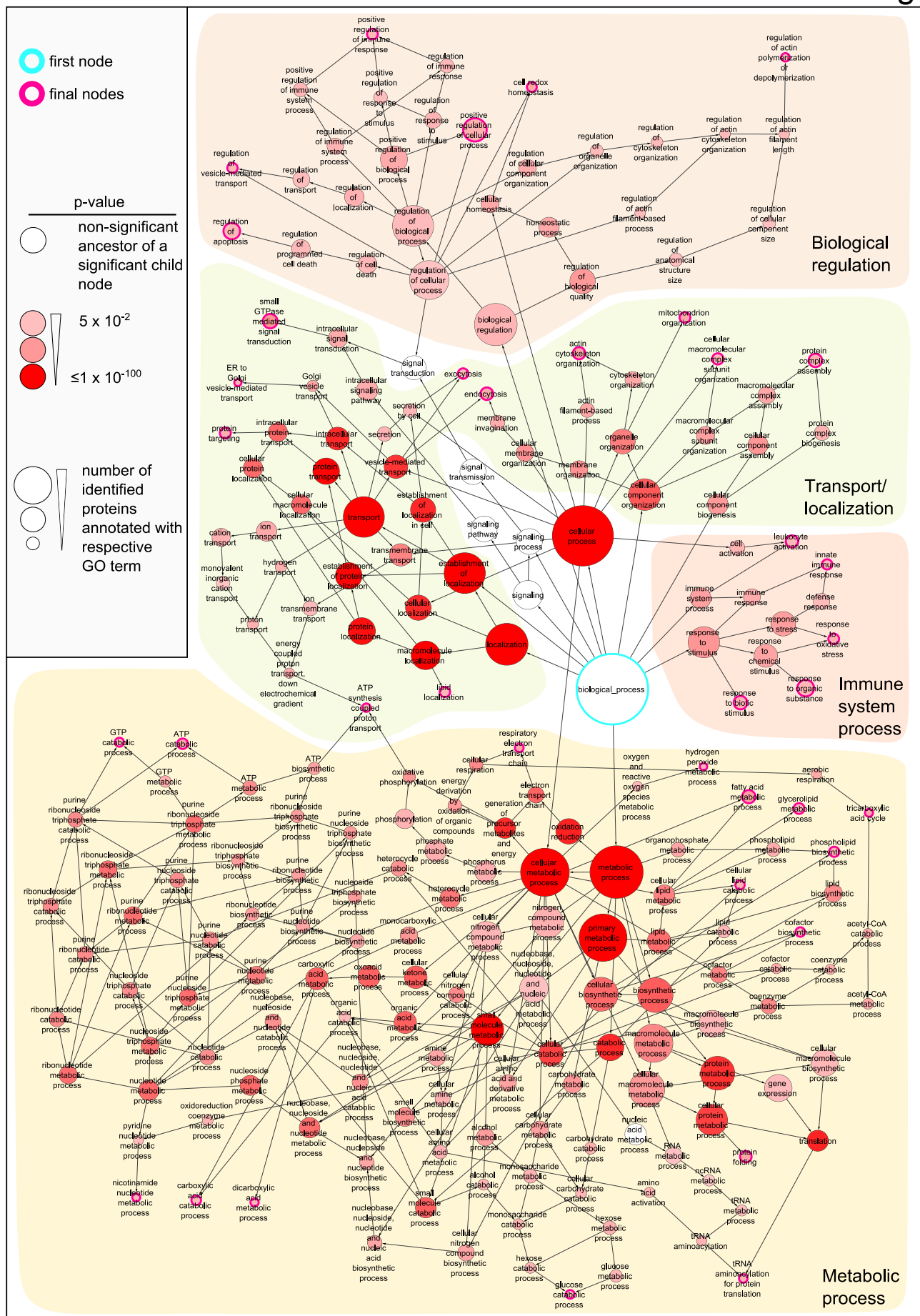

Supplement: S5 Fig — GO enrichment analysis for biological processes (BP) of the 2307 host proteins identified in untreated LCV samples using BiNGO (Cytoscape). Hierarchical structure, read from inside (first node, blue encircled) to outside (final nodes, pink encircled). Subnetworks of highly enriched biological processes are highlighted (metabolic process, transport/localization, biological regulation, immune system process). Significance cut-off value for visualization was set to 10−10, ancestor terms with p > 10−10 are depicted if final child term had p-value < 10−10. Tabular outline of whole analysis including exact p-values and full lists of proteins for each GO term can be found in S2 Dataset. (PDF) [file ppat.1005408.s005.pdf]

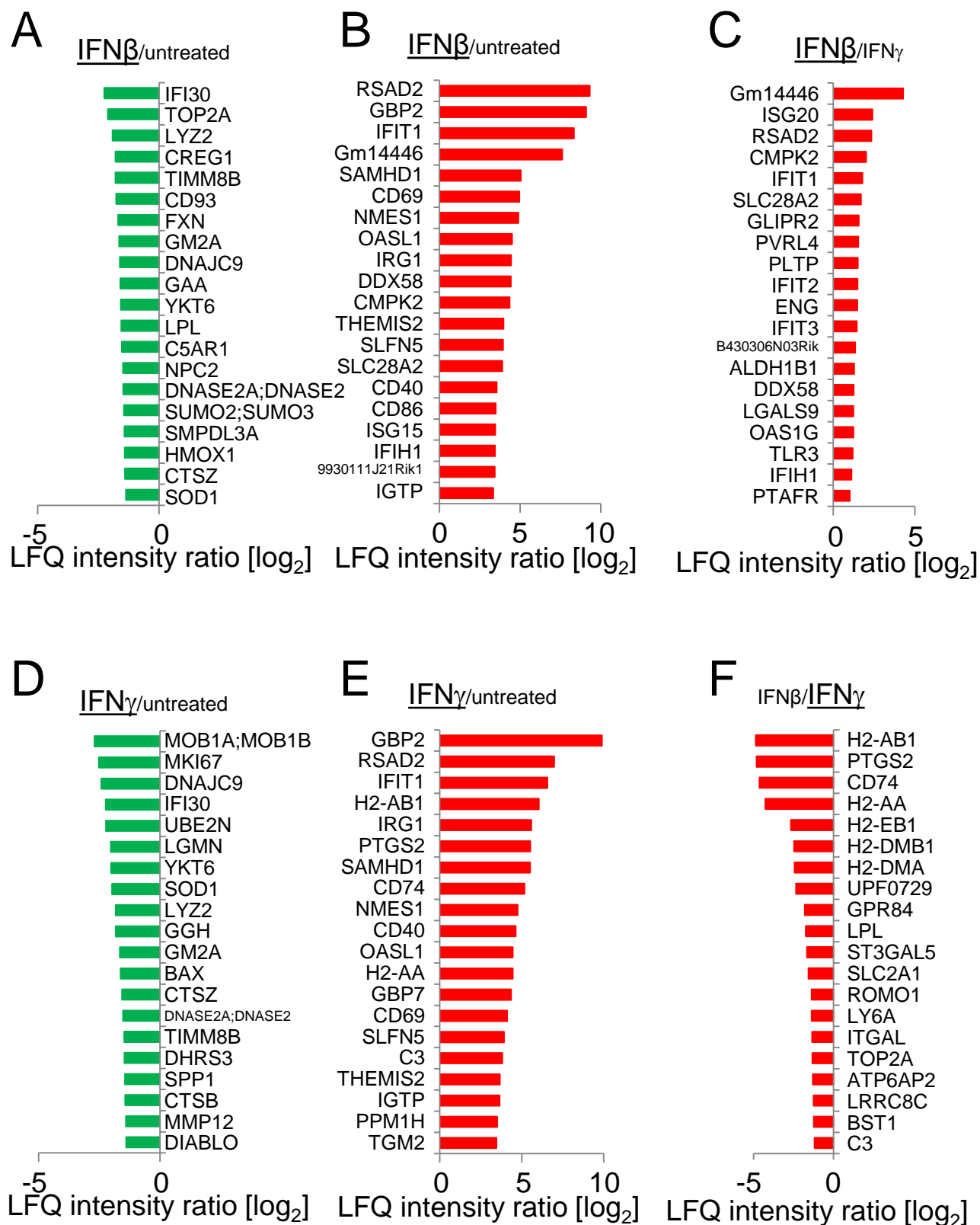

Supplement: S6 Fig — Quantitative proteomic analysis of LCVs isolated 2 h p.i. with L. pneumophila ΔflaA from BMMs left untreated or treated with 50 U/ml IFNβ or IFNγ 16–18 h prior to and during infection. Bar graphs show top 20 proteins with a significant higher (red) or lower (green) abundance at LCVs from IFNβ- (A, B) or IFNγ- (D, E) treated BMMs compared to untreated cells, and direct comparison of IFNβ- versus IFNγ-treated samples (C, F). Bar graphs correspond with volcano blots depicted in Fig 3C–3E. Proteomic analysis was done from 6 (untreated), 5 (IFNγ) and 4 (IFNβ) individual LCV isolations. (PDF) [file ppat.1005408.s006.pdf]

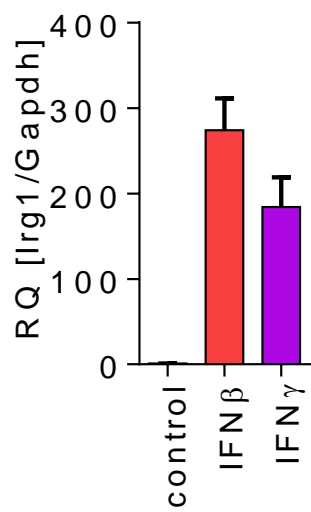

Supplement: S7 Fig — Irg1 gene expression in WT BMMs left untreated or treated with 50 U/ml IFNβ or IFNγ for 16–18 h was determined by qRT-PCR. Data are mean + s.e.m. of 2 independent experiments done in triplicates. (PDF) [file ppat.1005408.s007.pdf]

Fig S8

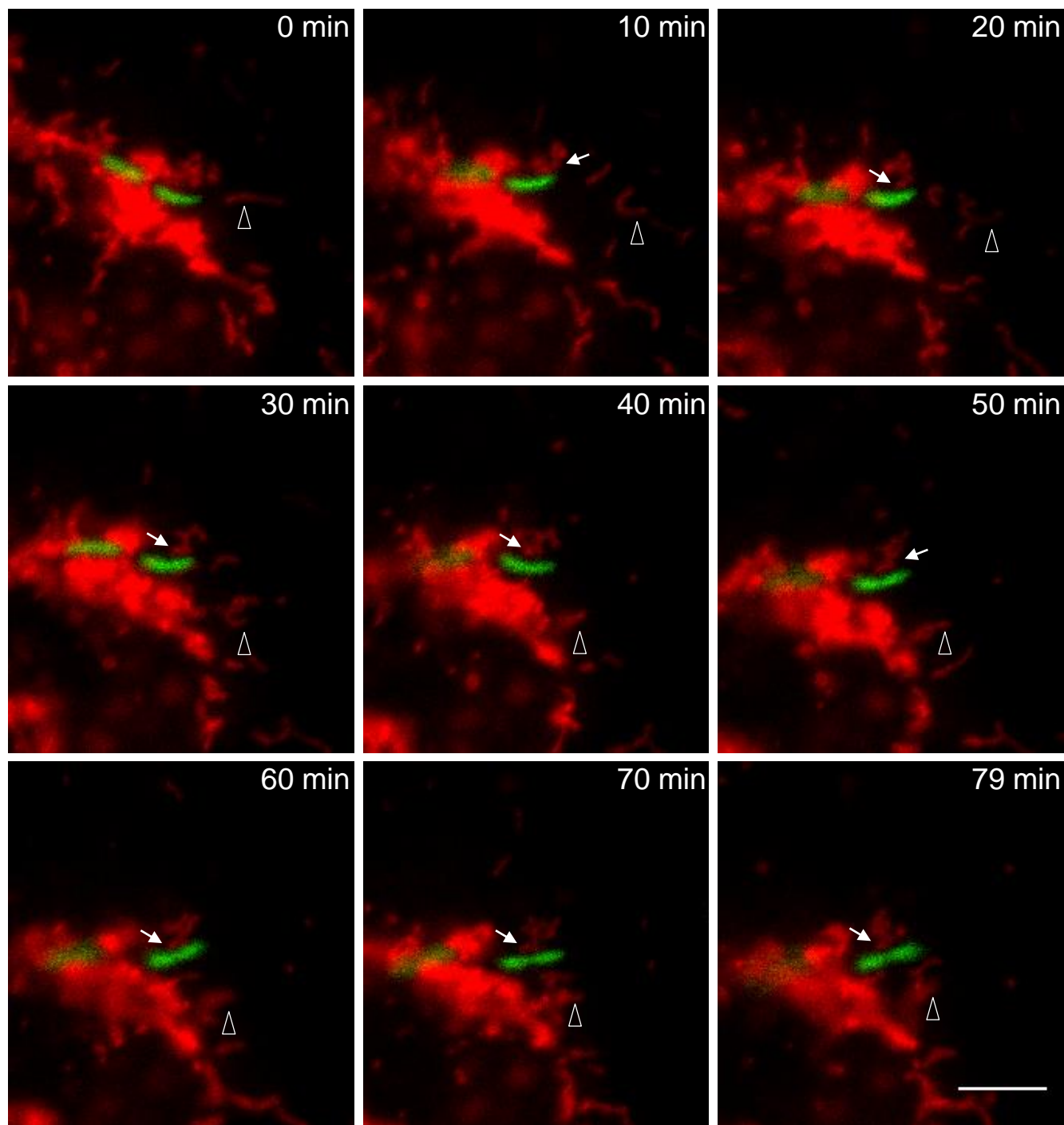

Supplement: S8 Fig — Representative frames from time-lapse confocal imaging of mitotracker stained (red) WT BMMs infected with L. pneumophila ΔflaA expressing eGFP (green). Imaging starts approximately 2 h p.i.. White arrow points toward a single mitochondrion staying in close proximity of the intracellular L. pneumophila, while other mitochondria move dynamically within the cell (open white arrowhead). The full sequence of frames with a 1-minute-time resolution can be found in S1 Video. (PDF) [file ppat.1005408.s008.pdf]

A

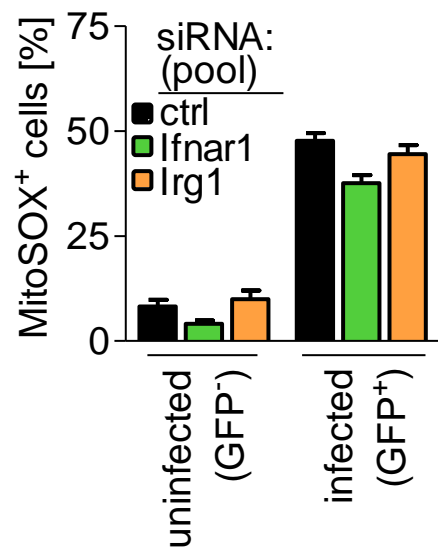

B

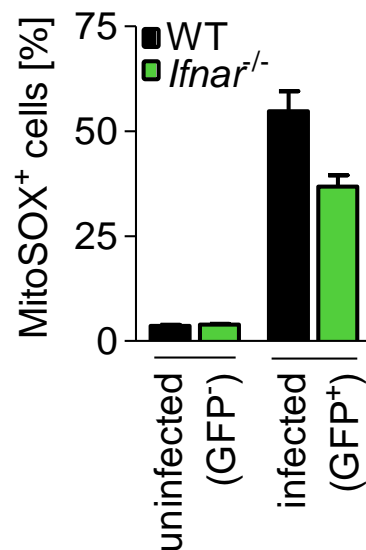

Supplement: S9 Fig — WT BMMs transfected with siRNAs 24 h prior to infection (A), or WT and Ifnar -/- BMMs (B) were infected with eGFP-expressing L. pneumophila wt and proportions of mitochondrial ROS producing (MitoSOX+) cells were determined by flow cytometry in infected (GFP+) and uninfected (GFP-) populations, respectively. Data represent mean + s.e.m. of 2 (B) or 4 (A) experiments done in triplicates. (PDF) [file ppat.1005408.s009.pdf]
