# Supplementary material for: IFNs Modify the Proteome of Legionella-Containing Vacuoles and Restrict Infection Via IRG1-Derived Itaconic Acid
Source: PLoS Pathog. 2016 Feb 1;12(2):e1005408. doi: 10.1371/journal.ppat.1005408 (PMC4734697; doi:10.1371/journal.ppat.1005408)
Supplement: S1 Table — (DOCX) [file ppat.1005408.s014.docx]

**S1 Table.**

| siRNAs | | | |
| --- | --- | --- | --- |
| target gene | siRNA ID |  |  |
| *Cxcl10* | s201504 |  |  |
| *Cxcl10* | s68048 |  |  |
| *Gbp2* | s66501 |  |  |
| *Gbp2* | s66503 |  |  |
| *Gbp3* | s79881 |  |  |
| *Gbp3* | s79882 |  |  |
| *Gbp7* | s106347 |  |  |
| *Gbp7* | s106348 |  |  |
| *Ifit1* | s68058 |  |  |
| *Ifit1* | s68059 |  |  |
| *Ifnar1* | s68085 |  |  |
| *Ifnar1* | s68086 |  |  |
| *Irg1*(#1) | s68386 |  |  |
| *Irg1*(#2) | s68387 |  |  |
| *Nmes1* | s119452 |  |  |
| *Nmes1* | s119453 |  |  |
| *Rsad2* | s81519 |  |  |
| *Rsad2* | s81520 |  |  |
| *Slfn5* | s116120 |  |  |
| *Slfn5* | s116121 |  |  |
| *Themis2* | s106616 |  |  |
| *Themis2* | s106617 |  |  |
