# Supplementary material for: IFNs Modify the Proteome of Legionella-Containing Vacuoles and Restrict Infection Via IRG1-Derived Itaconic Acid
Source: PLoS Pathog. 2016 Feb 1;12(2):e1005408. doi: 10.1371/journal.ppat.1005408 (PMC4734697; doi:10.1371/journal.ppat.1005408)
Supplement: S2 Table — (DOCX) [file ppat.1005408.s015.docx]

**S2 Table.**

| quantitative real-time PCR Taqman Assays | | | |
| --- | --- | --- | --- |
| target gene | Taqman assay ID |  |  |
| *Gbp7* | Mm00523797_m1 |  |  |
| *Ifit1* | Mm00515153_m1 |  |  |
| *Ifnar1* | Mm00439544_m1 |  |  |
| *Slfn5* | Mm00806095_m1 |  |  |
| quantitative real-time PCR primer sets | | | |
| target gene | forward primer sequence (5' - 3') | revers primer sequence (5' - 3') | probe sequence  (5'-FAM, 3'-TAMRA) |
| *Cxcl10* | CATCCCTGCGAGCCTATCC | GGATTCAGACATCTCTGCTCATCA | CCCACGTGTTGAGATCATTGCCACG |
| *Gapdh* | TGTGTCCGTCGTGGATCTGA | CCTGCTTCACCACCTTCTTGA | CCGCCTGGAGAAACCTGCCAAGTATG |
| *Gbp2* | GTTGAGAAGGGTGACAACCAGAA | TGGTTCCTATGCTGTTGTAGATGAA | CTGGATCTTTGCTTTGGCAGTCCTCCTC |
| *Gbp3* | AAGTCCTACCTCATGAATCGTCTTG | ACCCTTGGTTTCGGATTGC | AACCATGGCTTCTCCTTGGGCTCC |
| *Irg1* | AGGCACAGAAGTGTTCCATAAAGTC | AGTGAACAGCAACACCATTAACAAA | AGACTTCAGGCTCCCACCGACATATGCT |
| *Nmes1* | TCAACCCCAAAAGCTTATAACCA | CCCTCCGGACTTTTTGCA | CAACCAGCAATGGAAGCCCGTTG |
| *Rsad2* | TGGTGCCTGAATCTAACCAGAA | TCCACGCCAACATCCAGAAT | CGCTTTCTGAACTGTACCGGTGGCC |
| *Themis2* | TGATCCTAAAACCCCAGTATATGCT | GACGGGATCTTGACGATGCT | CAAGCCATCATGCACATGCGCA |
